# Genetic interaction network has a very limited impact on the evolutionary trajectories in continuous culture-grown populations of yeast

**DOI:** 10.1186/s12862-021-01830-9

**Published:** 2021-05-26

**Authors:** Joanna Klim, Urszula Zielenkiewicz, Marek Skoneczny, Adrianna Skoneczna, Anna Kurlandzka, Szymon Kaczanowski

**Affiliations:** 1 Department of Microbial Biochemistry, Institute of Biochemistry and Biophysics, Polish Academy of Sciences, Pawińskiego 5a, 02-106, Warsaw, Poland; 2 Department of Genetics, Institute of Biochemistry and Biophysics, Polish Academy of Sciences, Pawińskiego 5a, 02-106, Warsaw, Poland; 3 Laboratory of Mutagenesis and DNA Repair, Institute of Biochemistry and Biophysics, Polish Academy of Sciences, Pawińskiego 5a, 02-106, Warsaw, Poland; 4 Department of Bioinformatics, Institute of Biochemistry and Biophysics, Polish Academy of Sciences, Pawińskiego 5a, 02-106, Warsaw, Poland

**Keywords:** Compensatory evolution, Experimental evolution, Genetic interactions, Yeast, Genomics, Transcriptomics

## Abstract

**Background:**

The impact of genetic interaction networks on evolution is a fundamental issue. Previous studies have demonstrated that the topology of the network is determined by the properties of the cellular machinery. Functionally related genes frequently interact with one another, and they establish modules, e.g., modules of protein complexes and biochemical pathways. In this study, we experimentally tested the hypothesis that compensatory evolutionary modifications, such as mutations and transcriptional changes, occur frequently in genes from perturbed modules of interacting genes.

**Results:**

Using *Saccharomyces cerevisiae* haploid deletion mutants as a model, we investigated two modules lacking *COG7* or *NUP133*, which are evolutionarily conserved genes with many interactions. We performed laboratory evolution experiments with these strains in two genetic backgrounds (with or without additional deletion of *MSH2*), subjecting them to continuous culture in a non-limiting minimal medium. Next, the evolved yeast populations were characterized through whole-genome sequencing and transcriptome analyses. No obvious compensatory changes resulting from inactivation of genes already included in modules were identified. The supposedly compensatory inactivation of genes in the evolved strains was only rarely observed to be in accordance with the established fitness effect of the genetic interaction network. In fact, a substantial majority of the gene inactivations were predicted to be neutral in the experimental conditions used to determine the interaction network. Similarly, transcriptome changes during continuous culture mostly signified adaptation to growth conditions rather than compensation of the absence of the *COG7, NUP133* or *MSH2* genes*.* However, we noticed that for genes whose inactivation was deleterious an upregulation of transcription was more common than downregulation.

**Conclusions:**

Our findings demonstrate that the genetic interactions and the modular structure of the network described by others have very limited effects on the evolutionary trajectory following gene deletion of module elements in our experimental conditions and has no significant impact on short-term compensatory evolution. However, we observed likely compensatory evolution in functionally related (albeit non-interacting) genes.

**Supplementary Information:**

The online version contains supplementary material available at 10.1186/s12862-021-01830-9.

## Background

Genetic interaction networks are the subject of a growing field of scientific research—systems biology. In high-throughput studies the calculation of fitness epistasis has been employed to define interaction networks among gene deletions. The phenomenon of epistasis has been studied for over 100 years and its definition varies in time and between scientific fields. In this study it is considered in terms of the fitness effects of two mutations alone, or in combination, and it is used to describe situations in which those combinations show nonadditive phenotypic effects. Interactions between genes occur when the fitness of a double mutant differs from the additive expectation based on the individual fitness effects of the mutations. Epistasis is positive when mutations in two genes cause an organism to exceed the fitness predicted from individual effects of deleterious mutations on the organism, and it is negative when mutations cause the organism to fall below the predicted fitness. Genetic interactions identify functional relationships between genes and functional modules. Functional modules are groups of genes (related by genetic interactions) that participate in the same biological process.

Genetic interaction networks based on fitness epistasis also contain information regarding adaptive landscapes, which means that fitness data show whether in the background of a given deletion mutant a deletion of another gene is beneficial or deleterious. In the early 1920s, Ronald Fisher pioneered the notion that adaptation is, on the whole, a hill-climbing process. In other words, adaptation proceeds through a progressive accumulation of beneficial mutations [1]. Soon after, Sewall Wright proposed in a seminal paper that the relationship between genotypes could be visualized (or described) as a landscape [2]. The fitness is the “height” of the landscape, while the genotypes or phenotypes are points in the multidimensional space located beneath the landscape. During evolution, genotypes achieve local peaks of fitness by gradual step mutations. The evolutionary theories predict that different populations evolve toward a stable outcome unless the adaptive peak is constantly in motion i.e. landscape is unstable [3]. If a genetic interaction network is stable, it could be expected that the landscape resulting from that network has an impact on the trajectory of compensatory evolution in similar conditions and similar genetic backgrounds. The history of compensatory evolution studies is as old as microbial experimental evolution itself (for a review see [4]). Once a deleterious mutation is introduced in a given population, its negative effect on fitness can be alleviated through compensatory evolution. Thus, compensatory evolution is an adaptive process in which specific mutations are driven to fixation to suppress or mask the effect of a deleterious mutation.

In this paper, we investigated whether the genetic interaction network has an impact on the evolution of biological functional modules. We expected that loss of function of one component of such modules would lead to evolutionary reorganization of the cellular machinery. Simultaneously, we verified the impact of compensatory evolution on modules of genetic interactions. As an experimental model, we utilized the budding yeast *Saccharomyces cerevisiae*, which is the most thoroughly genetically characterized eukaryotic microorganism. In this model, the genetic interaction network is well characterized [5]. It has been shown that nonlethal deleterious mutations are widespread in populations [6, 7]. Two large-scale evolutionary studies suggest that such mutations are frequently compensated for by other mutations elsewhere in the genome [8, 9], a statement which is supported by other findings [10–13].

We tested the stability of two functional modules associated with two evolutionarily conserved genes with many interactions: *COG7*, which encodes a protein involved in vesicular transport, and *NUP133*, which encodes a protein engaged in nucleocytoplasmic transport. Using whole-genome sequencing and microarray techniques, we analyzed the evolutionary trajectory in *cog7Δ* and *nup133Δ* haploid mutants subjected to experimental evolution in continuous culture in a non-limiting minimal medium. Continuous cultures assure the stability of environmental conditions and the metabolic state of cells whereas a chemically-defined medium ensures reproducibility of results. Moreover, a defined medium minimizes the number of variables thus facilitating the exploration of those important for the given study. This is particularly advantageous when whole transcriptome studies are performed, which is one of the elements of this research [14, 15].

We came to the conclusion that the supposedly compensatory inactivation of genes in the evolved strains was very rarely in accordance with the established fitness landscape and only a few changes in the transcriptomes of the evolved yeast populations were in keeping with predictions. A substantial majority of the inactivations were in fact predicted to be neutral on the basis of the landscape of the interaction network [16]. However, we identified mutations in genes that are functionally related to *COG7* or *NUP133* and in new genes that are likely involved in adaptations to continuous culture and medium conditions.

## Results

### Experimental evolution—general description

In order to check whether the genetic interaction network has an impact on evolution of biological functional modules, we performed two sets of experiments. The evolving yeast strains were devoid of either the *COG7* or the *NUP133* gene. These evolutionarily conserved genes [17–20], were selected because *cog7Δ* and *nup133Δ* mutants exhibited enhanced fitness while combined with some other deletion mutants in the high throughput Synthetic Genetic Array (SGA) assay of genetic interaction networks [16]. According to the BioGRID repository (https://thebiogrid.org [21]), both genes exhibit numerous genetic interactions: *COG7*—352 and *NUP133*—530. Based on the BioGRID database statistics available for *S. cerevisiae* S288C strain, there are 443,432 non-redundant genetic interactions among the 5,962 unique yeast genes, therefore each gene exhibits ca. 74 genetic interactions, on average. According to data from Costanzo et al*.* [16], *COG7* is involved in 248 and *NUP133* in 362 interactions in comparison to 165 on average. Taken together, these data imply that *COG7* and *NUP133* genes participate in 1.5- to sevenfold more genetic interactions than the average gene. Thus, the likelihood of observing compensatory mutations arising in other genes seemed high even in a short-term experiment.

The *COG7* gene encodes an element of the oligomeric Golgi (COG) complex. The COG complex organizes core vesicular trafficking to and within the Golgi apparatus and is also involved in protein glycosylation. It is remarkable for the number of interactions, and a lack of nearly any of its elements significantly affects numerous cellular processes [22]. The Cog7 subunit forms a binary complex with Cog5 [23]. Their interaction is conserved from yeast to humans and its disruption causes glycosylation defects [24]. Nup133, a component of the nuclear pore complex (NPC), is an element of the coat nucleoporin subcomplex (CNC) [25, 26]. Nup133 has multiple functions mainly linked to RNA export and NPC assembly [27, 28]. It is also involved in the functioning of the mitotic spindle by recruiting centromeric CENP-F to NPCs in the prophase and maintaining the association of the centrosome with the nuclear envelope at mitotic entry [29].

We also performed a second set of experiments designed to speed up the evolution, in which double mutants *cog7Δ msh2Δ* and *nup133Δ msh2Δ* were used. The lack of *MSH2* gene increases the frequency of mutations and we assumed that introducing *msh2Δ* into *cog7Δ* or *nup133Δ* could increase the rate of appearance of beneficial variants by increasing the dynamics of mutation rate.

Before the continuous culture experiments were run, growth rates of knockout strains were measured in comparison to the wild-type strain in the non-limiting minimal medium used in further studies (Additional file 1). As expected, all mutants had increased doubling times (0.4–23.6%) in comparison to the wild-type.

**Fig. 1:**
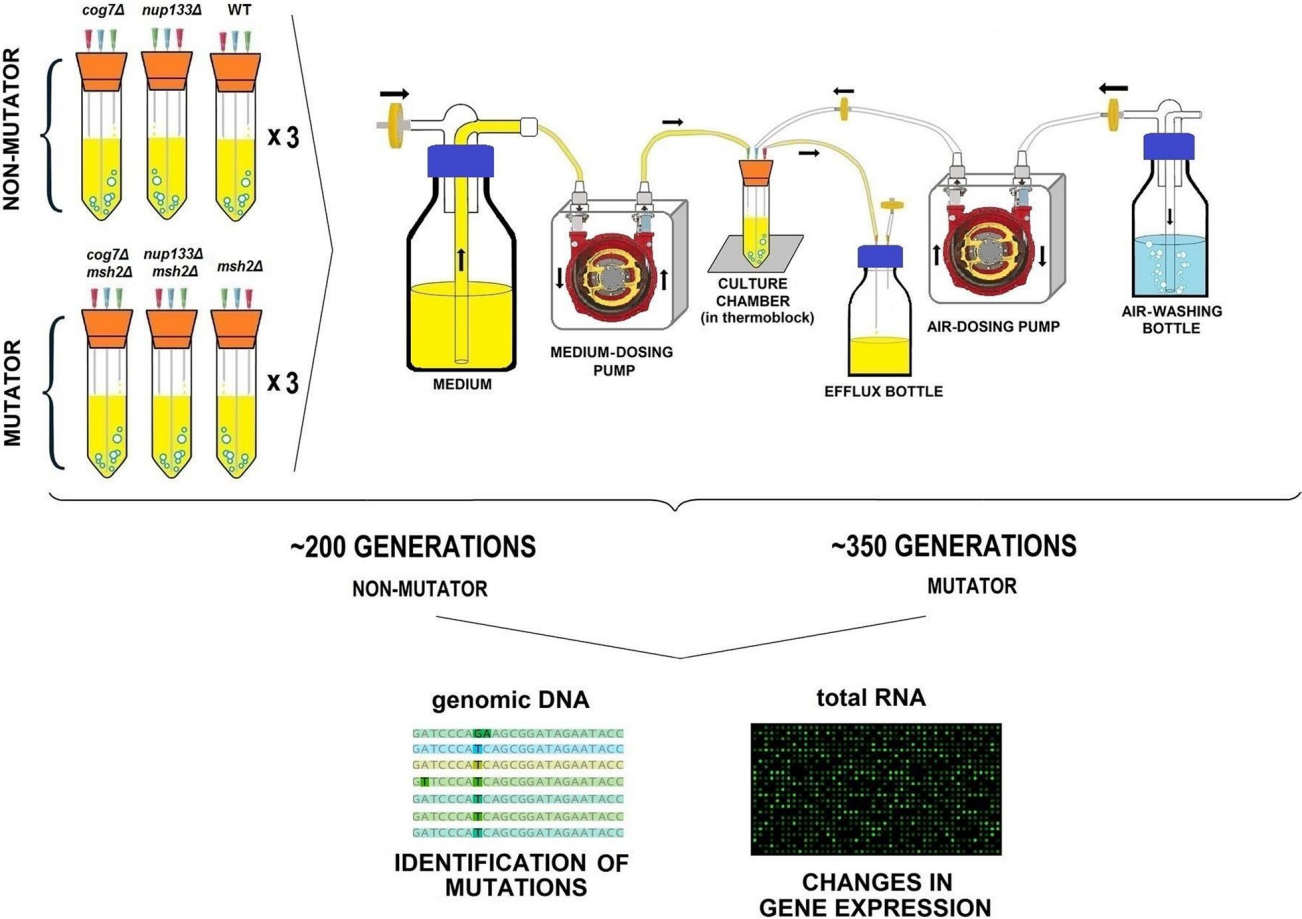
Schema of the continuous culture evolution experiments. This figure was in part composed of elements derived from: https://commons.wikimedia.org/wiki/File:Bredel_Werkingsprincipe_animatie.gif, https://commons.wikimedia.org/wiki/File:Microarray_and_sequencing_flow_cell.svg (modified)

The studied strains were cultivated in parallel with three replicates for each strain, resulting in 12 independent continuous cultures. To detect adaptations unrelated to the presence of the *cog7Δ* or *nup133Δ* deletions, we performed similar evolutionary experiments for control strains; the parental W303 strain and the *msh2Δ* strain, both in triplicate. The cell culture density was maintained in a stable state of 5.2 ± 1.1 × 10^7^ cells per mL, the density equivalent to the late exponential phase of batch culture in the same medium. The experiments were ended due to the appearance of flocculation. All non-mutator populations (*MSH2*) were propagated for approximately 200 generations, and the mutator (*msh2Δ*) ones for ca. 350 generations (Fig. 1). Then, the genomes and transcriptomes of evolved populations were determined.

Because it was reported that in some evolutionary experiments, yeast cells can switch their ploidy ([30–32], for a review see [33]), we evaluated the ploidy of evolved populations by DNA content analyses using flow cytometry and we found that haploidy was distinctly dominant in all populations. Analyses were performed for all biological replicates, and representative DNA content profiles are shown in Additional file 2.

### Mutations in evolved yeast populations

**Fig. 2:**
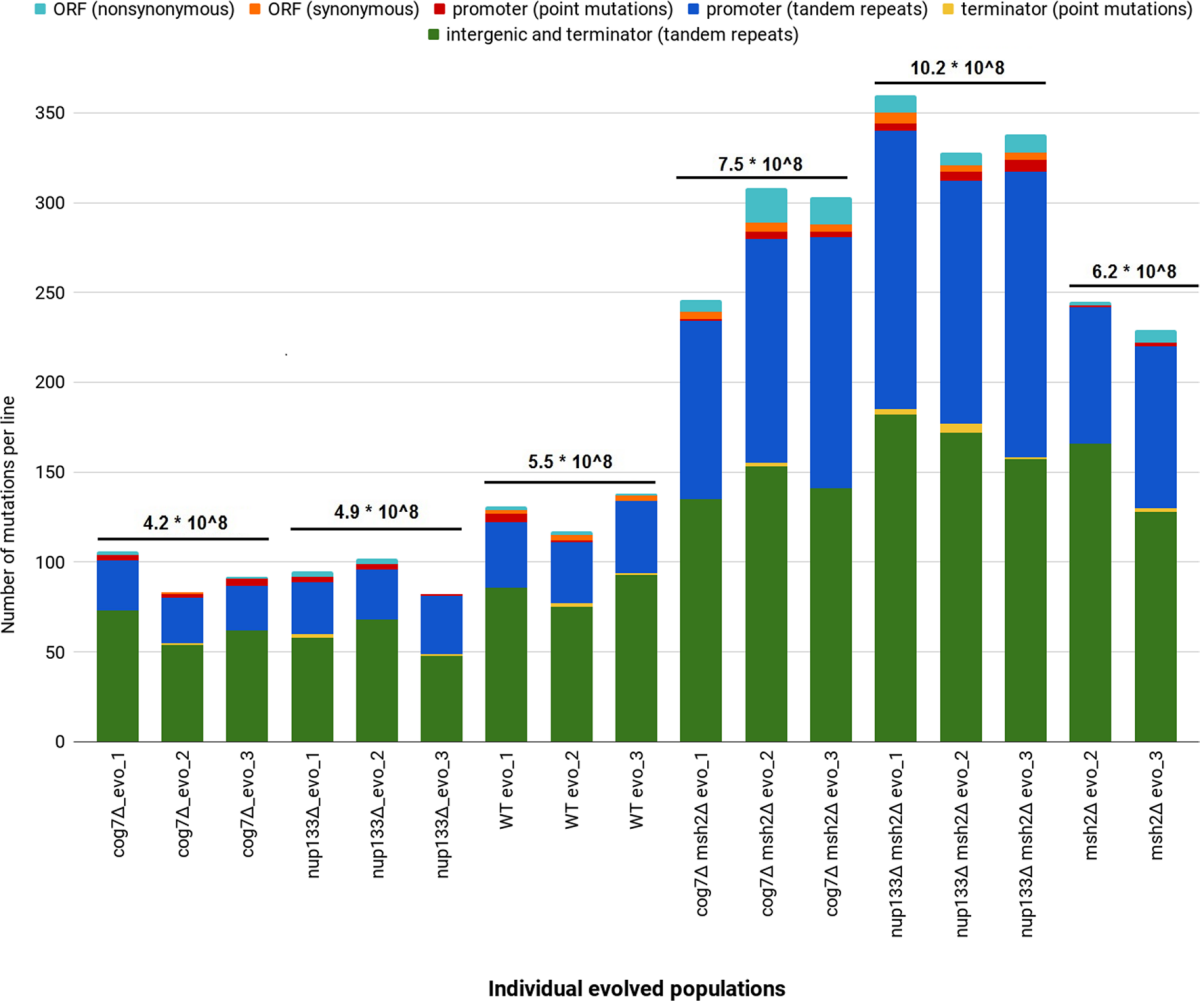
Graphical summary of DNA-sequencing data of 17 evolved yeast populations including a number and type of mutations per each evolved yeast population and average fixed mutation rate per genotype (shown above the bars). Fixed mutation rate was defined as an average number of mutations (with a frequency threshold ≥ 50%) identified in each yeast strain, per base, per replication cycle (mut/nuc/rep). The type of mutation is represented by color: light blue = nonsynonymous, orange = synonymous, red = promoter (point mutations), navy = promoter (indels located in tandem repeats), yellow = terminator (point mutations), green = intergenic and terminator (indels located in tandem repeats)

To identify the genetic changes in the evolved populations complete genomes of 17 independently evolved populations (one of the evolved *msh2Δ* population could not be processed due to technical reasons) were sequenced and compared with the genomes of corresponding ancestor strains sequenced de novo. The average coverage for a population whole-genome sequencing across all samples was 113×. Variants supported by less than 10 reads across a given population were removed. To filter for high-frequency variants, a 50% mutation frequency threshold (on a population scale) was applied. Figure 2 shows summarized data obtained for each population studied. At the scale of whole populations, we identified ca. 100–130 new mutations in non-mutator strains and ca. 200–350 mutations in the mutator ones. Next, we calculated fixed mutation rate that was defined as an average number of mutations (with a frequency threshold ≥ 50%) identified in each yeast strain, per base, per replication cycle (mut/nuc/rep). The fixed mutation rates were similar in the *cog7Δ, nup133Δ*, and WT strains. As expected, the fixed mutation rates of the control *msh2Δ* and the *cog7Δ msh2Δ* and *nup133Δ msh2Δ* strains were higher than in non-mutator ones. The vast majority of all mutations were localized to tandem repeats (TRs), and numerous were found in putative promoters (Fig. 2). For one mutation in an ORF, there were on average ten (non-mutators) or eight (mutators) mutations in promoter regions.

In further analyses we only considered single nucleotide polymorphisms (SNPs) and short insertion/deletion events. Intergenic mutations and those identified in TRs regions were not included. Because there were only small differences in the number and types of mutations between different biological replicates for a given yeast strain, they were combined for further analysis. Point mutations in open reading frames (ORFs), putative promoter (5′ upstream regions—up to 500 nucleotides), and terminator regions (3′ downstream regions—up to 250 nucleotides) were analyzed separately. Summarized data are presented in Table 1 and Additional file 3: Table S3,  Additional file 4: Table S4 and Additional file 5: Table S5 that show detailed information.

**Table 1:** Characteristics of point mutations identified in evolved yeast strains

	Mutations					
Strain	ORFs		Putative promoter regions		Putative terminator regions	
	Total	Genotype-specific^b^	Total	Genotype-specific^b^	Total	Genotype-specific^b^
*cog7Δ*	4	1 (1^a^)	2	1	2	1
*nup133Δ*	5	3 (3^a^)	2	1	6	5
WT	4	3 (2^a^)	4	3	4	3
*cog7Δ msh2Δ*	44	43 (36^a^)	8	8	3	2
*nup133Δ msh2Δ*	36	36 (24^a^)	15	15	8	7
*msh2Δ*	8	7 (7^a^)	3	3	2	2

^a^Nonsynonymous

^b^Unique to given strain in terms of given experimental group (non-mutator or mutator)

Data combined for three biological replicates. Intergenic mutations and those identified in TRs regions not included. Mutation frequency threshold ≥50%.

In the evolved *cog7Δ* and *nup133Δ* populations we found few unique nonsynonymous substitutions, one (in *MEC1*) in *cog7Δ* population 3, and three (in *CAN1*, *PFK27*, and *WHI2*) in *nup133Δ* population 1 and 2. According to the Costanzo et al*.* [16] databases neither *MEC1* interacts with *COG7* nor *CAN1*, *PFK27* or *WHI2* with *NUP133*. A similar number of unique mutations was detected in the evolved wild-type populations, where we identified mutations in *MSY2, URE2,* and *DAL82*, of which only two were nonsynonymous (Table 1 and Additional file 3: Table S3).

In the evolved *cog7Δ msh2Δ* populations, 43 unique mutations were identified in ORFs, and 36 of them alter the amino acid sequence of encoded proteins (Table 1 and Additional file 4: Table S4). In the evolved *nup133Δ msh2Δ* populations, 36 unique ORF mutations were identified, of which 24 were non-synonymous (Table 1 and Additional file 4: Table S4).

In the *cog7Δ msh2Δ* populations, the set of genes bearing nonsynonymous mutations could be divided into six subgroups according to their biological function (except for two uncharacterized ORFs, YPL247C and YPL277C), shown in Table 2 (for more details, see Additional file 6), however, no enrichment of gene ontology (GO) terms was found. In the *nup133Δ msh2Δ* strain mutated genes belonged to seven functional groups (Table 2, for details, see Additional file 7). Again, no GO term enrichment was found.

**Table 2:** Gene ontology classification of mutated genes according to their biological meaning

Biological meaning	*cog7Δ msh2Δ*	*nup133Δ msh2Δ*
Cell wall and cell division	*DMA2, ELM1, SKM1*	*DMA1, GIC1, SCW4*
Cellular metabolism	*AMF1, DAK2, GLN1, GNA1, HXK1, ICL1, SEO1, SHM2, SHP1, SOL4, TKL1*	*CAN1, CEX1, EFT2, SAM2*
Endocytosis	*ART5, ECM21*	
Genome maintenance	*AMN1, EXO5, HUR1, IRR1, NET1, PIF1, SAP30*	*APC5, GIC1, MEC3, RIM15, RSC1, SWI1*
Golgi/ER-associated	*HSP104, KRE5, PMR1, TRX2*	*PRK1, SLA2*
Histone modification		*ACC1, BRE1*
Peroxisome-associated		*INP1, PEX21*
Protein folding		*EMP65, HSP60, IBA57, TAH1*
Transcription, translation	*ACE2, MRPS5, PAB1, PRP2, RPL4B, SSN3, WAR1*	

It should be noticed that *cog7Δ msh2Δ* and *nup133Δ msh2Δ* populations share some enriched groups e.g., cellular metabolism, and genome maintenance.

In the control *msh2Δ* evolved strain only a small number of uniquely mutated genes was found: *BRR2, DRN1, MDN1, PMD1, RPL42B, URE2,* and *YBT1* thus GO enrichment analysis was not performed.

### Putative adaptive mutations in evolved strains

We verified whether the loss-of-function mutations acquired during our continuous culture experiments fit in the yeast genetic network described by Costanzo et al*.* [16], which has been used as a model of the fitness landscape. Such mutations are likely to have a universal beneficial impact on the deletion mutants subjected to the experiments. We assumed that the loss of function of a second gene has a statistically significant impact on the fitness of the original deletion mutant if the absolute value of the difference between the fitness of the single and the double deletion mutant is higher than three times the standard deviation of the fitness of the double mutant. Among changes present in the evolved mutant strains there were few causing nonsense mutations leading to protein truncation caused by premature stop codons. One such mutation (in *WHI2*) was found in the *nup133Δ* population 1, but according to Costanzo et al*.* [16] inactivation of *WHI2* has no impact on fitness of *nup133Δ*. No putative loss-of-function mutations were found in *cog7Δ* populations. In the double mutant *cog7Δ msh2Δ* three putative loss-of-function mutations were found, *ACE2* (in population 3), *ECM21* and *SAP30* (in population 2), and three in *nup133Δ msh2Δ*, *MEC3, RIM15* and *TAH1* (in population 3). According to Costanzo et. al. [16], inactivation of *ACE2* and *ECM21* has no impact on fitness of neither *cog7Δ* nor *msh2Δ*, while inactivation of *SAP30* is deleterious both for the *cog7Δ* and *msh2Δ* mutants. Loss of function of *MEC3* has no impact on the fitness of *nup133Δ* and *msh2Δ* while inactivation of *RIM15* is beneficial and inactivation of *TAH1* is deleterious when combined with *msh2Δ.* In the control *msh2Δ* evolved strain only the *URE2* gene was truncated (population 2). Ure2 participates in nitrogen catabolite repression [34]. Deletion of *URE2* has no impact on the fitness of *msh2Δ* in the genetic interaction network.

Thus, the supposedly compensatory inactivation of genes in the evolved populations followed the expectation (i.e., was in accordance with landscapes derived from known genetic interactions [16]) only one in eight cases. In five cases no impact on mutant fitness was predicted, and in two cases gene inactivation might cause fitness decrease. Thus, a substantial majority of the inactivations were in fact predicted to be neutral.

What is more, we did not observe any trend of mutations accumulation in genes that participate in the same functional module as primarily deleted *COG7*, *NUP133* or *MSH2*, respectively (documented in Table 3). Also, the number of genes showing positive and negative interactions with the tested genes was similar (data not shown).

**Table 3:** A number of genotype-specific genes mutated in the course of evolution exhibiting genetic interactions (GIs) with primarily deleted *COG7*, *NUP133* or *MSH2*, respectively

Primarily deleted gene	Evolved strain	Genes exhibiting genetic interaction/s with primarily deleted gene		Mutated genes
		Lenient^a^	Stringent confidence^b^	
*COG7*	*cog7Δ*	0	0	2
	*cog7Δ msh2Δ*	5 (1)	0	51 (8)
*NUP133*	*nup133Δ*	0	0	4
	*nup133Δ msh2Δ*	8 (4)	3 (1)	51 (15)
*MSH2*	*cog7Δ msh2Δ*	6	1	51 (8)
	*nup133Δ msh2Δ*	3	2	51 (15)
	*msh2Δ*	0	0	10 (3)

^a^(P < 0.05)

^b^(P < 0.05 and GIS > 0.16 or < -0.12)

The analysis was based on Costanzo et al. [16] genetic interaction network data and two filtration thresholds were applied. Only genes mutated in ORFs and promoter regions (in parentheses) were analyzed.

Thus, we did not find correlations between neither *MSH2, COG7* nor *NUP133* and mutated genes in terms of genetic interactions. The identified genes are not the parts of the same modules as originally deleted ones and they do not correlate with the fitness landscape of genetic interaction network. Thus, the fitness landscape does not seem to govern the process of evolution of tested deletion mutants.

The genetic interaction network we based our study on was determined for one specific growth condition and one *S. cerevisiae* strain. It is quite likely that upon different conditions different interactions will be revealed [35]. However, since a significant conservation of GIs and GI network structure has been reported even between distant yeast species [36, 37], it seems less likely that it would completely change the overall image of the network itself.

To understand the mechanisms of adaptation of studied mutants we searched through all genes mutated in coding sequences hoping to identify unanticipated mutations. In result, we identified mutations in genes functionally related to *COG7* or *NUP133.* We also identified mutations in genes involved in adaptations to the chemostat conditions, identified by other researchers. Since our experimental continuous culture conditions resembled a chemostat, we could expect such changes. They are briefly described below.

In the evolved *cog7Δ* population we found the mutation leading to a change in the Mec1 protein. In *nup133Δ* – mutations leading to changes in Can1, Pfk27, Whi2. These proteins play important roles in DNA repair, sugar metabolism, and general stress response but are not known to be, directly or indirectly, linked to *COG7* or *NUP133*, respectively. In the evolved *cog7Δ msh2Δ* and *nup133Δmsh2Δ* populations we selected a few putative compensatory mutations. The characteristics of the respective proteins and amino acid changes that resulted from identified mutations are given in Table 4. In *cog7Δmsh2Δ* we found mutations in *PMR1* and *TRX2* (population 2), likely related to *COG7*. We assume that mutations in these genes, if they enhanced the activity of the encoded proteins, could compensate for the *COG7* loss. Pmr1 protein delivers calcium and manganese ions to the Golgi. Both ions are required for proper processing and trafficking of polypeptides through the secretory pathway [38]. It has been reported that Pmr1 plays a role in the sorting of secreted proteins at the trans-Golgi network/late Golgi membranes and in glycosylation since *PMR1* inactivation led to some perturbations of the glycosylation [39]. What is more, according to the data deposited in the BioGRID, *PMR1* inactivation combined with deletion of some of the COG genes causes severe growth defects. Therefore, it seems that the Pmr1 and Cog proteins are closely related functionally. Trx2 is an element of the thioredoxin system that plays an essential role in cell protection against oxidative stress induced by reactive oxygen species (ROS) and participates in cell repair. Interestingly, the Trx2 protein is also required for vacuole inheritance and ER-Golgi transport where it participates in the retrieval of resident ER proteins cycling between the Golgi and the ER [40]. Therefore, it can be postulated that Trx2 has an impact on the Golgi-ER communication and could alleviate the vesicle trafficking defects caused by a lack of Cog7. In the *nup133Δmsh2Δ-*derived populations, we found five mutated genes that might be functionally related to *NUP133.* Four of them: *ACC1* (population 2)*, BRE1, RSC1* (population 3) and *SWI1* (population 1) encode proteins engaged in NPC stabilization, transcription and chromatin silencing [41–44]. Because Nup133 is also involved in these processes [45–47], it is likely that the mutated genes could, to some degree, compensate for the *NUP133* loss. The fifth protein, Cex1, interacts directly with NPCs and participates in the tRNA export [48]. Thus, the mutation in *CEX1* (identified in population 3) is likely to compensate for some functions compromised in *nup133Δ*.

**Table 4:** Putative compensatory mutations in evolved *cog7Δ msh2Δ* and *nup133Δ msh2Δ* mutator populations

Primarily deleted gene	Mutated gene	Function	aa change	aa position	PFAM Domain (aa)	Motif (ELM prediction)	Putative connection with deleted gene
*COG7*	*PMR1*	Ion transporting ATPase	F → C	926	PF00689 P-type ATPase, transmembrane domain (762–934)		Required for Ca^2+^ and Mn^2+^ transport into Golgi
	*TRX2*	Cytoplasmic thioredoxin	M → V	40	PF00085 Thioredoxin domain (10–100)		Required for ER vesicle fusion with the Golgi
*NUP133*	*ACC1*	Regulates histone acetylation; required for synthesis of long-chain fatty acids that were proposed to stabilize the NPC [41]	H → R	2068	PF01039 Acetyl-CoA carboxylase (1574–2130)		Nup133 also plays a role in transcription regulation and chromatin silencing
	*BRE1*	Required for methylation of selected histones [42]	L → S	599			
	*RSC1*	Regulates nucleosome positioning and transcription regulation [43]	A → V	98	PF00439 Bromodomain (31–98)		
	*SWI1*	Regulates transcription by remodeling chromatin [44]	T → N	15		GSK3 phosphorylation recognition site	
	*CEX1*	Component of nuclear tRNA export pathway [48]	A → T	468			Associates with NPCs by interacting with Nup116p

Putative compensatory mutations in evolved *cog7Δ msh2Δ* and *nup133Δ msh2Δ* mutator populations

Because most of the amino acid changes were located in domains or motifs important for the function of the respective proteins it is likely that they caused significant functional changes. We suggest that the mutations identified in the above genes could be compensatory by alleviating the fitness loss due to the deletion of *COG7* or *NUP133*. The missense mutations, by enhancing growth of evolved mutants, may facilitate the restoration of their fitness. However, the fact that not all evolved populations of a given strain carry such mutations suggests that alternative mechanisms of fitness compensation were achieved, or other compensatory events took place.

The majority of mutations occurred in genes involved in basic cellular processes. It is well known that culturing in a chemostat, which our experiments emulated (constant growth conditions) except for the absence of nutrient limitation, often results in altered cellular strategies independent of the original genotype but rather associated with the response to the environment (for a review see [49]). In our study, they are likely represented by changes not related to the original deletion. These changes may hinder connecting genotype to the observed mutations, but nonetheless, they provide valuable information about the spectrum of mutations that are adaptive in a specific environment. They also validate the conducted experiment indicating that it was carried out long enough to allow adaptation to growth conditions.

In the non-mutator strains, we identified mutations in the genes *HOG1* and *WHI2* linked before to the adaptation to chemostat conditions [50]. The Hog1 protein plays an essential role in multiple stress conditions (for a review see [51]) and Whi2 is essential for general stress response associated with cell cycle arrest [52]. We found nonsynonymous mutations (both frameshift and missense) in *HOG1* in wild-type (population 1 and 2) and *cog7Δ* (population 1) (Additional file 3). One of them resulted in a stop codon, the second mutation caused E186K substitution. In *cog7Δ* the mutation leading to R290K substitution was identified. In the gene *WHI2* missense and nonsense mutations occurred in *nup133Δ* populations, i.e., mutation causing substitution Q387* in population 1 and W273L in population 2 (Additional file 3). The presence of these mutations, likely inactivating Hog1 and Whi1, indicates that experimental conditions did not trigger stress responses. At present, we cannot explain putative advantageous qualities of the observed changes.

Mutations in *URE2* (WT population 2), *PFK27* (*nup133Δ* population 1), *BUL1* (*cog7Δ* population 1, and *nup133Δ* population 2), and *MSY1* (WT population 1 and 3) are likely to be adaptive. These genes encode proteins engaged in fructose metabolism (*PFK27*), regulation of nitrogen utilization (*URE2*), aa-tRNA synthesis (*MSY1*), and amino acid uptake (*BUL1*). Thus, in the non-mutator strains, the adaptations indicate that the environment caused a stronger selective pressure than did the introduction of gene deletions.

In the mutator populations, two genes related to continuous culture adaptation were affected: *RIM15* in *nup133Δ msh2Δ* (population 3), and *ACE2* in *cog7Δ msh2Δ* (population 3). Rim15 protein activates transition to the G0 phase in response to starvation [53], and its mutations have been described in a variety of nutrient-limited chemostat experiments [50, 54–57]. *ACE2* encodes a cell cycle-regulated transcription factor [58]. What is more, mutations in *ACE2* likely contribute to the evolution of aggregation phenotypes [59]. Altogether three genes engaged in cell cycle regulation (*ACE2, WHI2* and *RIM15*) and associated with the adaptation to growth conditions were mutated.

Besides the above mutations, we found other potentially growth conditions-adaptive ones, in genes related to basic metabolism. It is likely that the mutations identified in *PRO3* and *URE2* (*msh2Δ* and wild-type), *AMF1, DAK2, GLN1, GNA1, HXK1, ICL1, PRO3, SEO1, SOL4* and *TKL1 (cog7Δ msh2Δ*) and *PEX21* and *SAM2* (*nup133Δmsh2Δ*) could have beneficial effects on yeast growth in continuous culture in non-limiting minimal medium (for details see Additional file 6 and Additional file 7).

To sum up, we did not find compensatory gene inactivations linked to *COG7* or *NUP133*. Our genomic data indicate that mutations do not tend to accumulate in genes belonging to a given functional module. Instead, we identified a few putative compensatory mutations related to *COG7* or *NUP133* and compensating for their loss, but not included in the Costanzo et al. [16] genetic interaction network. Substantially more mutations were linked to general adaptations to growth conditions.

### Parallel evolution in the evolving yeast populations

The data from repeated experiments were pooled to facilitate the analyses discussed above. However, this approach prevented us from detecting mutations shared by different populations evolved from the same ancestor, i.e., cases of parallel evolution. Thus, in the next step, we re-examined ORF mutations in each population separately. Table 5 shows that in the non-mutators at least two populations which evolved from the same genotype shared mutations in one (*WHI2—*in *nup133Δ*) or two (*MSY1* and *HOG1*—in wild-type) genes. As already mentioned, mutations in *WHI2* are speculated to provide a fitness advantage under certain environments or in combination with other compensatory mutations [8, 60] whereas a role of mutations in *HOG1* remains unclear.

Although in the *msh2Δ* populations more mutations occurred, as expected, they were not more reproducible between replicates than in the non-mutator ones. The only shared mutations in *cog7Δ msh2Δ* populations were in *SEO1* encoding putative transmembrane transporter and in *CDC3* encoding a component of the septin ring (Table 5). This indicates that parallel evolution within a given yeast strain was overall rare in the evolving yeast populations.

Interestingly, we identified four genes that were mutated more than once across all yeast populations i.e., *BUL1* (α-arrestin, a component of the Rsp5p E3-ubiquitin ligase complex), *PRO3* (catalyzes the last step in proline biosynthesis), *URE2* (glutathione peroxidase involved in regulating cellular nitrogen utilization), and already mentioned *HOG1*. Mutations in these genes might be beneficial in our experimental conditions, regardless of genetic background.

### Transcriptomic changes in evolved populations

In parallel with the whole population sequencing of evolved yeast cell populations, we performed transcriptome analyses of the same samples. Here, we aimed to reveal if the transcriptome changes are directly linked to mutations in 5’ upstream regions. We envisaged that changes in gene expression levels within the continuously cultured cell population may have compensatory characteristics or even that some of the differentially expressed genes (DEGs) belong to *COG7* or *NUP133* interaction modules.

To reject changes in gene expression occurring due to rapid shift in conditions at the onset of continuous culture, we compared the transcriptomes of the cell population samples harvested after about 35 generations, i.e., shortly after reaching the steady-state, with the transcriptomes of final, adapted populations after about 200 generations (for WT, *cog7Δ* and *nup133Δ*) or after about 350 generations (for *msh2Δ, msh2Δ cog7Δ* and *msh2Δ nup133Δ*). Agilent Yeast v2 microarray and Cy3 Cy5 two-color labeling were employed in this experiment (see Materials and Methods for more details). Hereafter, for brevity, we call the results of these comparisons as adaptive transcriptomes.

First, we analyzed the similarities between adaptive transcriptomes of individual biological replicates within each genotype and between genotypes. We employed Euclidean distance as a measure of this similarity and the results are shown in Additional file 8, A and B.

This analysis revealed slightly higher level of similarity between adaptive transcriptomes of biological replicates of WT and *cog7Δ* genotypes than between those of the remaining genotypes, but in general these differences were not statistically significant (p-value calculated with Student t-test was between 0.44 and 0.81). The similarities between averaged transcriptomes of each genotype (Additional file 8, B) were on the same level. The similarity tree, shown in Additional file 8, C, revealed a somewhat stronger relationship between biological replicates of the WT genotype and that of *cog7Δ* genotype than the remaining ones. Thus, it seems that evolutionary trajectories of WT and *cog7Δ* populations were convergent, while the remaining ones were more distinct. However, in general, the prevalent similarity between replicates of a given genotype in combination with transcriptomic data abundance prompted us to pool data gathered for individual replicates to show strain-specific changes. Complete adaptive transcriptome results (see Additional file 9) were subject to several analyses.

Having found the differences of adaptive transcriptomes between genotypes, as the next step we performed the gene ontology (GO) analysis of identified genes that were differentially expressed between genotypes. This analysis allowed us to determine the functions of these genes and to assess the potential compensatory capacity of the observed changes in their expression. A given gene was considered as differentially expressed if its expression had changed at least twofold (log2ratio ≥ 1, or ≤ − 1) in at least two of three biological replicates. The microarray data were validated for several DEGs by RT-qPCR giving excellent correspondence of the two sets of results (Additional file 10).

We found significant changes in the mRNA level for 7.7% of genes in the evolved wild-type, 6% in the evolved *cog7Δ* and 2.9% the evolved *nup133Δ* and for the mutator strains: 9.4% in *msh2Δ*, 4.6% in *cog7Δ msh2Δ,* and 4.3% in *nup133Δ msh2Δ.* The results (summarized in Additional file 11) were then analyzed in the context of the interaction network fitness landscape with the assumption that downregulation of gene expression can mimic its loss-of-function (Table 5).

**Table 5:**
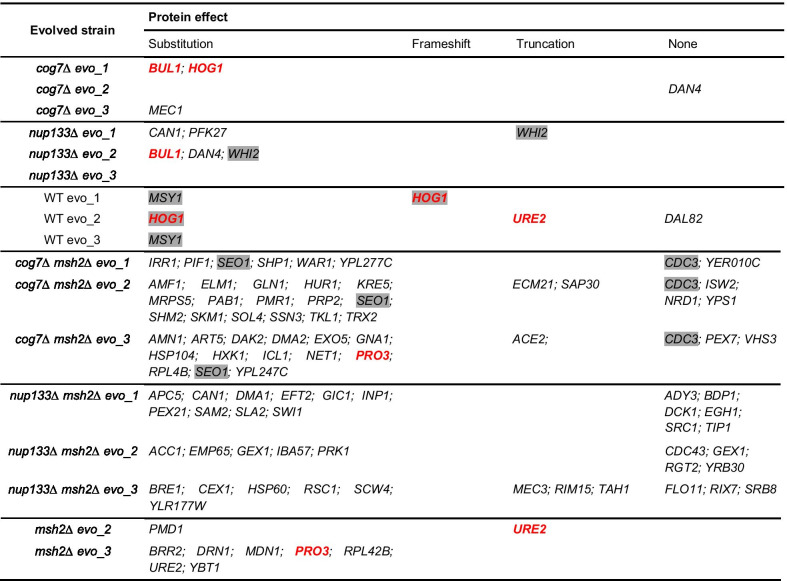
Mutations in ORFs of evolved non-mutator and mutator populations, grouped according to their predicted effect

Genes mutated in more than one biological replicate of a given strain are highlighted in gray, and genes mutated more than once across all tested strains are in red

Table 5 shows the impact of the genes exhibiting altered expression levels during compensatory evolution on the fitness of the deletion mutants assayed according to the genetic interaction network fitness landscape. Notably, upregulation was more common than downregulation for those genes whose inactivation was predicted to have a negative impact on fitness. For instance, in the evolved *cog7Δ* strain, out of the total of 57 DEGs whose absence should be deleterious according to the fitness landscape data as many as 45 were upregulated. Moreover, according to evolutionary theories, the level of fixation of deleterious mutations is very low. Thus, it could be expected that mutations leading to down-regulation of genes with a predicted deleterious effect of inactivation are very rare. In contrast to this expectation, we observed that still such event is quite frequent (12 of 57).

**Table 6:** Gene expression changes in evolved yeast strains vs. fitness landscape

Impact in the interaction network		Downregulated	Upregulated
Evolution of *cog7Δ*			
On cog7Δ**	Deleterious	12	45
	Beneficial	6	2
Evolution of *nup133Δ*			
On *nup133Δ*	Deleterious	7	11
	Beneficial	3	3
Evolution of *msh2Δ*			
On msh2Δ**	Deleterious	14	68
	Beneficial	24	10
Evolution of *msh2Δ cog7Δ*			
On cog7Δ**	Deleterious	15	62
	Beneficial	9	4
On msh2Δ**	Deleterious	14	68
	Beneficial	21	10
Evolution of *msh2Δ nup133Δ*			
On *nup133Δ*	Deleterious	4	9
	Beneficial	6	4
On *msh2Δ*	Deleterious	13	19
	Beneficial	14	9

**Statistically significant (p-value < 0.01) under-representation of deleterious genes that were down-regulated (according to the Fisher exact test)

### Genetic interactions vs. transcriptomic changes

Next, we verified changes in expression levels of the genes encoding components of the molecular machines of which the Cog7 and Nup133 proteins are a part. We checked if there is any enrichment in downregulated genes exhibiting positive GIs or, conversely, an enrichment in upregulated genes exhibiting negative GIs with *COG7*, *NUP133* or *MSH2.* Only genes exhibiting positive or negative GIs with *COG7*, *NUP133* or *MSH2* were analyzed. However, we did not find enrichment in genes having genetic interactions among DEGs (Table 7).

**Table 7:** Percentage of DEGs in evolved strains showing genetic interactions (GI) with the *COG7*, *NUP133* or *MSH2* gene

Evolved strain	DEGs exhibiting genetic interactions with (%)		
	*COG7*	*NUP133*	*MSH2*
	In accordance with functional module logic		
*cog7Δ*	3.3	2.6	0.8
*nup133Δ*	0.0	4.4	0.5
wild-type	1.2	2.5	0.6
*cog7Δ msh2Δ*	0.0	1.5	0.3
*nup133Δ msh2Δ*	1.4	0.0	0.5
*msh2Δ*	0.6	4,0	1.2
No. of genes exhibiting GIs with deleted gene	175	204	62

Results of analysis based on Costanzo et al*.* [16] data when stringent confidence filtration threshold (P < 0.05 and GIS > 0.16 or < − 0.12) was applied

Moreover, even when the DEGs from the *cog7Δ* and *cog7Δ msh2Δ* are analyzed for interactions with the *NUP133* gene, or those from *nup133Δ* and *nup133Δ msh2Δ* for GIs with *COG7*, or those not bearing *MSH2* deletion for GIs with *MSH2*, the results show no difference from a random distribution, indicating a lack of correlation between the expression changes and genetic interactions with the original deletion. Similar results were obtained for DEGs of wild-type strain which additionally confirm that evolution of gene expression seems to be independent of genetic interactions. The outcomes of this analysis remain similar regardless of the Costanzo et al*.* [16] data filtration threshold applied [stringent confidence or lenient (data not shown)].

### Gene expression profiles in evolved strains—general descriptions of transcriptomes

At first, to find out whether the mutations identified in the 5′ upstream putative promoter regions directly affected transcription we compared the results of whole-genome sequencing of yeast populations with the observed gene expression changes. We found that there was no apparent correlation between promoter mutations and changes in transcript abundance (Additional file 12).

**Fig. 3:**
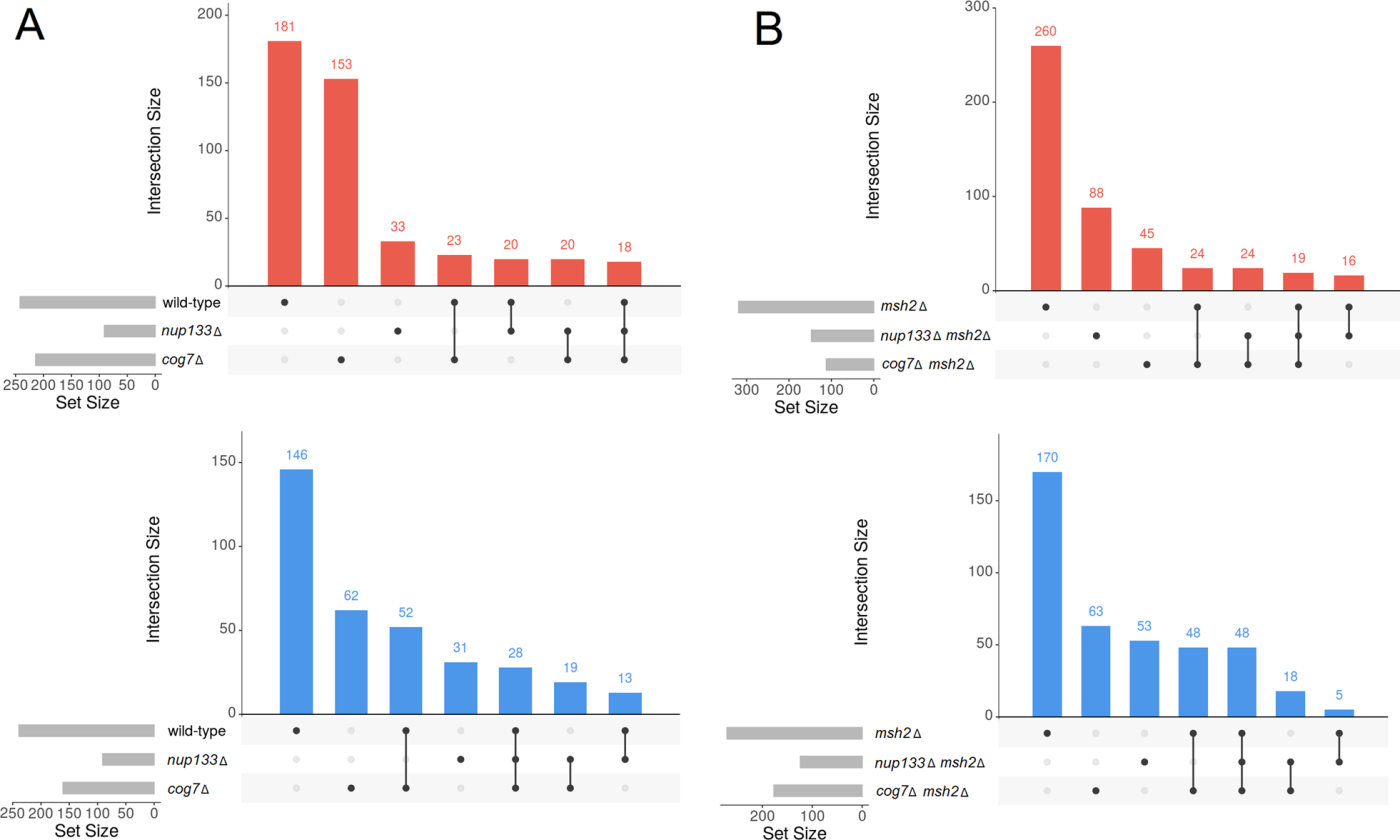
UpSet diagrams for genes whose expression was significantly up- (red) and downregulated (blue) in the three evolved non-mutator (**A**) and mutator (**B**) yeast strains. The horizontal bars show the number of differentially expressed genes of a given yeast strain, while the vertical bars display the size of sets of genes uniquely altered (on the left), followed by those exclusively shared between multiple pairwise comparisons. For more details concerning genes whose expression was significantly up- and downregulated in biological replicates of given yeast genotype see Additional file 13

Next, to find the biological significance of the transcriptomic changes we performed further analyses. To identify DEGs unique to each evolved strain the list of DEGs for each of the mutants and control strains were compared with each other, separately for the down- and upregulated DEGs, and separately for the *MSH2* and *msh2Δ* strains (Fig. 3).

**Fig. 4:**
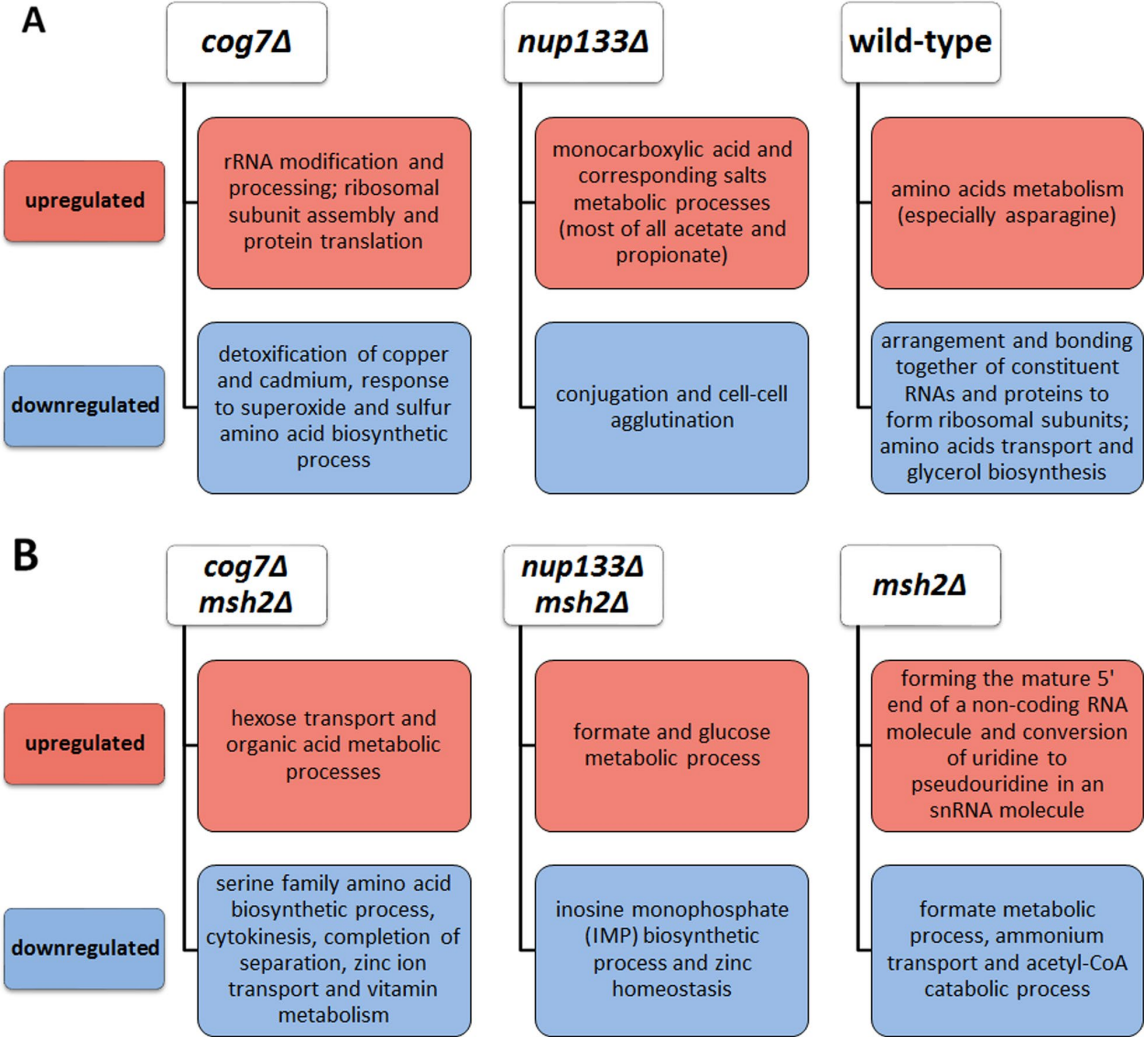
Enriched GO terms in the biological process category among DEGs from evolved non-mutator (**A**) and mutator (**B**) strains

Next, the lists of genotype-specific DEGs were subjected to a GO term enrichment analysis. The results are summarized in Fig. 4, and detailed information concerning top enriched GO terms of DEGs of all strains studied is provided in Additional files 14 and 15.

To sum up, we found that the transcriptomic changes in the evolved strains were mainly associated with adaptations to the culture conditions (e.g., downregulation of genes linked to the biosynthesis of glycerol and amino acids, and upregulation of genes linked to glucose uptake). We did not observe evident changes that might indicate significant alterations in the level of transcription of the genes within *COG7* or *NUP133* functional modules.

### Comparative analysis of evolved strains at the transcriptome level

**Fig. 5:**
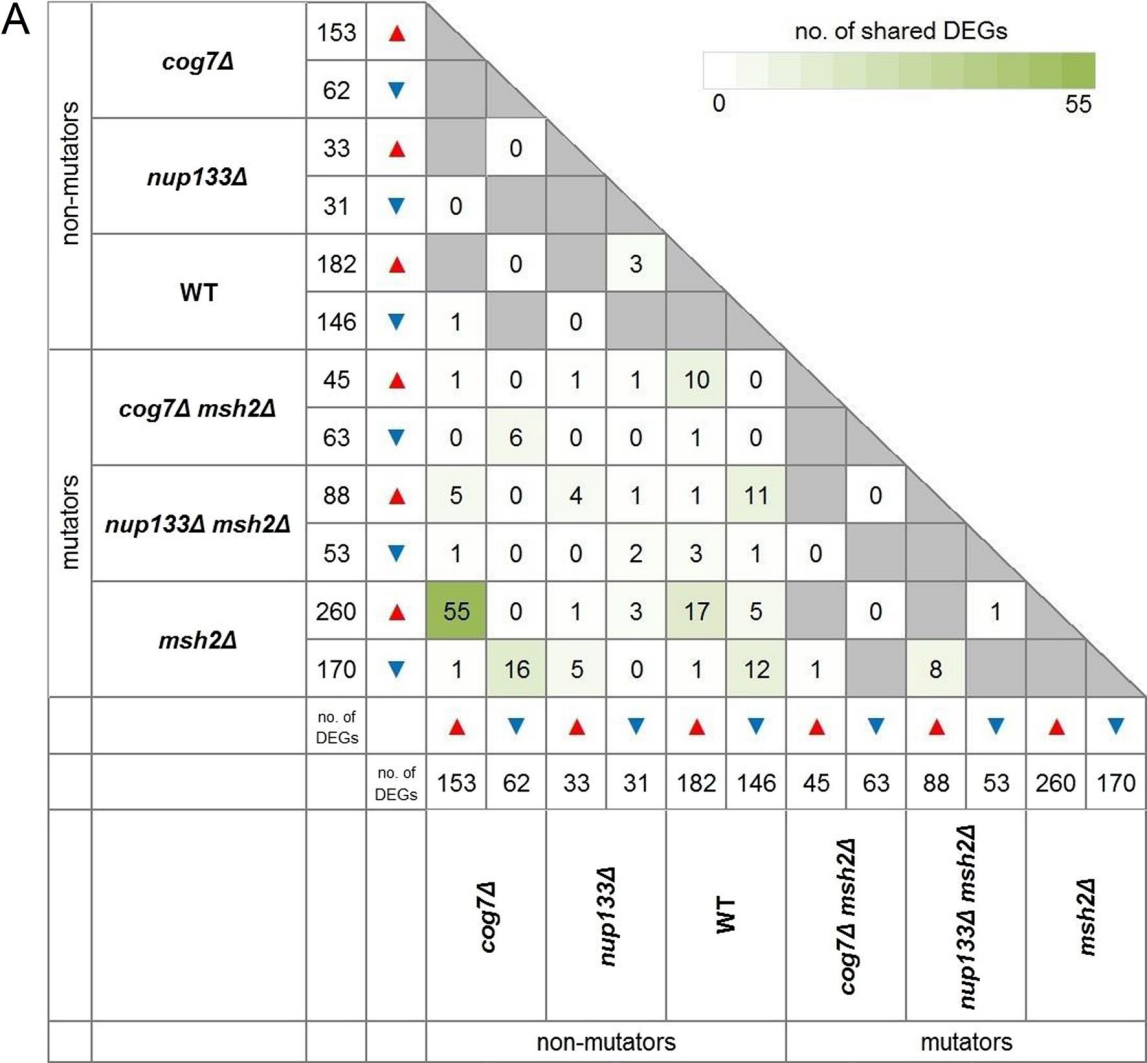

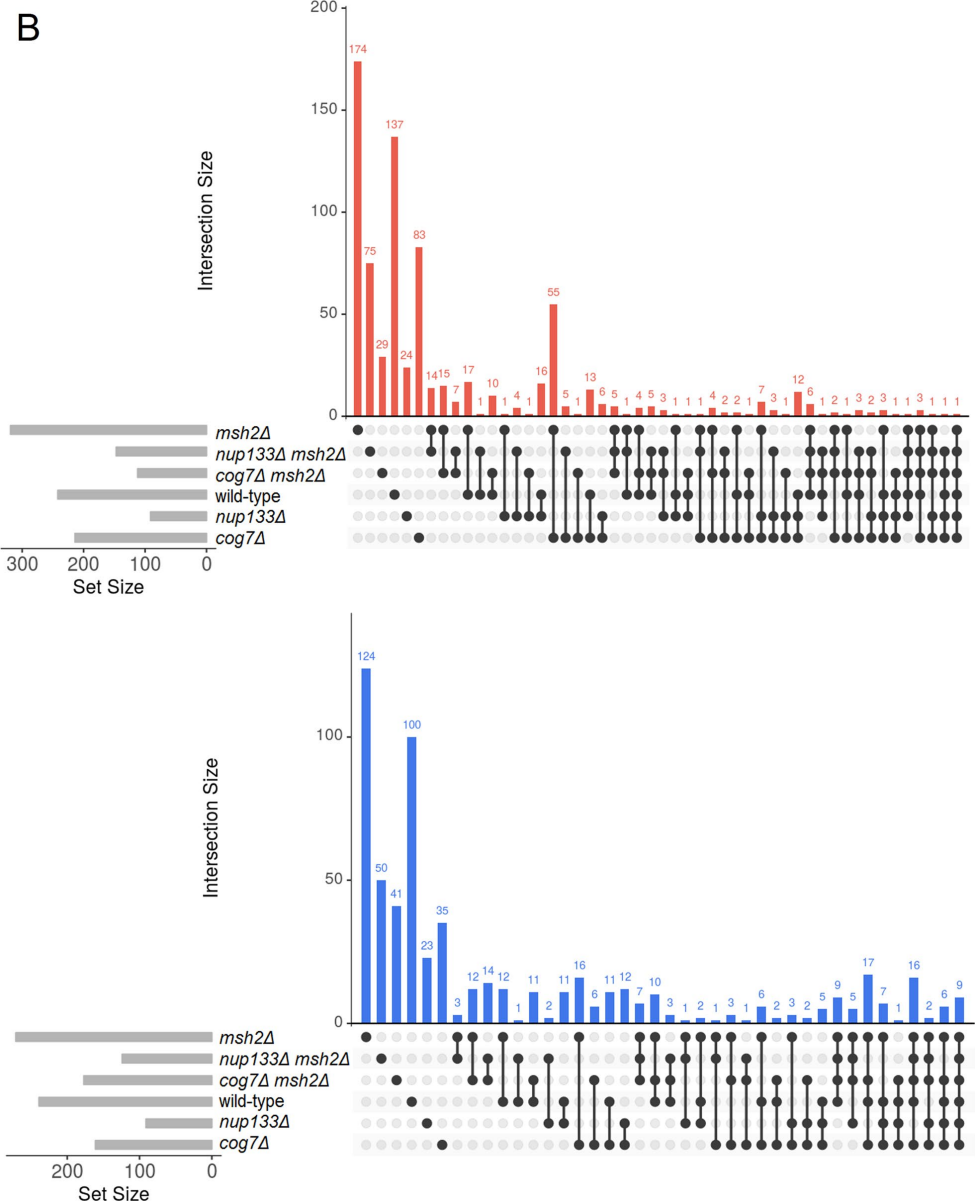
**A** DEGs shared by non-mutator and mutator strains. DEG sets unique to a given evolved strain (as determined within the non-mutator (*MSH2*) and mutator (*msh2Δ*) set of strains) were compared with one another. Grey cells depict pairs that a priori cannot have common DEGs. Triangles pointing upwards and downwards indicate up- and downregulated DEGs. **B** Detailed comparison of all yeast strains tested. UpSet diagrams indicate intersections between up- (red) and downregulated (blue) genes in all yeast strains tested, generated by multiple comparisons. The horizontal bars show the number of differentially expressed genes of a given yeast strain, while the vertical bars display the size of sets of genes ordered by increasing degree of intersections

To identify a common set of genes that could be differentially expressed in response to the *COG7* or *NUP133* deletion, unique DEGs of single mutants were compared with those from corresponding double mutants (Fig. 5). *cog7Δ* shared seven DEGs with *cog7Δ msh2Δ*, and *nup133Δ* shared six DEGs with *nup133Δ msh2Δ* in total.

The comparison of the transcriptomic changes in the evolved *msh2Δ* and *cog7Δ* strains has interesting outcomes. In total 71 DEGs are common for these two strains, of which 16 genes are down and 55 upregulated (the similarity of the upregulated DEG sets is reflected in the GO analyses). We noticed that the majority of the upregulated DEGs common to these two strains are associated with rRNA modifications and processing as well as with ribosome biogenesis. This is likely a metabolic adjustment to constant substrates availability and invariable growth conditions. However, it is not clear why these two mutants show such similar transcriptomic changes.

To sum up, functional analysis showed a different picture of changes in non-mutator and corresponding mutator strain. Hence, we did not observe sets of gene expression profiles of which can qualitatively assess the common response of the yeast strains to a given gene deletion.

## Discussion

The primary topic investigated in this study was re-examining the assumption that during evolution, nonlethal deleterious mutations are frequently compensated for by other mutations elsewhere in the genome. An earlier study of compensatory evolution following gene deletion in yeast by [8] indicated that the fitness losses caused by deletions are rapidly compensated for by mutations elsewhere in the genome. In particular, frequent compensation through loss-of-function nonsense mutations was observed. Echenique et al. reached a similar conclusion after studying 37 gene deletion mutants [9]. These researchers also identified a number of premature stop codons in other genes, which likely cause loss of function. In fact, approximately 20% of all non-silent mutations identified in the two above studies were putative loss-of-function mutations. These observations complemented other evolutionary experiments with microorganisms (bacteria and yeast), which have demonstrated that the most common adaptive changes are due to the loss or modification of a preexisting molecular function [32, 60–66].

The difference between our study and those of others is the scale. Specifically, we subjected two yeast mutants to very detailed examinations, whereas others performed large-scale experiments. We tested strains devoid of the genes *COG7* or *NUP133* because these genes are prolific interactors, and it was reasonable to expect fairly frequent beneficial inactivation(s) of elements of their networks. The cellular processes in which *COG7* and *NUP133* are involved are completely unrelated to each other; therefore, any evolutionary pattern found common to the two mutants was likely to be universal. Moreover, since the Cog7 and Nup133 proteins are evolutionarily conserved from yeast to humans, our findings should be valid for other organisms, as well, and our putative findings could be important in many biological aspects, including medicine. However, neither *cog7Δ* nor *nup133Δ* had been tested previously; thus, we could not compare our results to subsets of large data sources.

Notably, our study indicated that the lack of *COG7* or *NUP133* has an almost negligible impact on elements of modules of genetically interacting genes and on the transcriptional activity of other genes in the modules. Whether this result is a particular case to *COG7* and *NUP133* or whether the other studies identified only a specific set of modules has not been determined. This study also suggests that the fitness landscape of the genetic interaction network has some impact on the course of compensatory evolution. However, this impact can be observed mainly in transcriptomic experiments.

At the genomic level, we observed putative adaptations to the continuous culture environment, although in our experiments an element of nutritional stress (typical to chemostat) was omitted. This included mutation in the *WHI2*, *RIM15*, *ACE2* and *HOG1* genes which are related to environmental adaptations. Evidently, continuous culture conditions exert a stronger selective pressure than does a gene knockout itself.

Moreover, because three independently evolved populations derived from a given strain were sequenced, we could search for parallel evolutionary events. Surprisingly, we found only few parallel mutations which indicates that compensatory evolution is not deterministic and can lead to genetic divergence of evolving populations. What is more, the analysis allowed identification of recurrent mutations in *BUL1*, *PRO3*, *URE2*, and *HOG1* genes apparently beneficial in our experimental conditions regardless of the genetic background.

We also observed a few mutations that might compensate for the gene deletions assayed (Table 4). We hypothesize that changing the activity of these genes could restore the fitness of *cog7Δ* and *nup133Δ*. However, these changes are probably related to different mechanisms of fitness compensation or different compensatory events, since they constituted only individual cases and were not repeatable between different biological replicates for a given yeast strain.

The mutations identified in the present study comprised nonsense mutations causing protein truncation and likely depriving of activity, and missense mutations, which could have diverse effects on proteins: enhancing or inhibiting their activity or even modifying their functions and the outcome of such mutations is difficult to predict. However, we found that the majority of missense mutations occurred in genes involved in basic cellular processes. It is not clear why the adaptive landscape associated with these genes is not stable.

We also attempted to determine whether the adaptive landscape reflecting the yeast genetic interaction network is universal and has an impact on the evolution of yeast deletion mutants. We assumed that if the fitness landscape of the genetic interaction network governs the process of compensatory evolution, the loss-of-function mutations should also be beneficial for a given deletion mutant in the fitness landscape. This observation is supported by studies of the *WHI2* gene, which was also mutated in one of our experiments. It was shown that the landscape of the deletion of this gene is very unstable. According to the network of Constanzo et al., deletion of *WHI2* is neutral or slightly deleterious. This finding was confirmed in the study of Szamecz et al*.* [8], who found that the *WHI2* deletion is very weakly deleterious; these researchers also found a compensatory effect of *WHI2* inactivation during evolution of a *rpb9Δ* strain. In contrast, in other environmental conditions (in dense colonies and upon nutrient deprivation), deletion of *WHI2* is beneficial [67]. In unstable landscapes, loss-of-function mutations of *WHI2* are frequently observed [68].

In microevolution studies, the analysis of transcriptome is often the only method used to identify adaptive changes occurring in cell populations [69–72] and only very few studies analyzed changes at both the genome and transcriptome level [55, 73]. Because the reduction of the level of a given mRNA can, to some extent, mimic the effect of the lack of a gene, we carefully examined transcriptomic data. We observed fairly frequent downregulation of genes whose inactivation is predicted to be deleterious according to the fitness landscape of the genetic interaction network. However, these instances of downregulation were underrepresented, which suggested a tendency of the gene expression changes during continuous culture evolution to follow the fitness landscape. Nevertheless, similar to gene mutations, numerous cases of gene downregulation did not conform to the prediction obtained from the known pattern of genetic interactions. Thus, although our experimental approach may have some limitations, this result suggests that the fitness landscape is unstable and may be easily modified by genetic and external factors.

## Conclusions

Our results indicate that the assumption that nonlethal deleterious mutations are frequently compensated for by other mutations in genetically interacting genes during evolution is not universal and, in fact, does not include *COG7* and *NUP133* yeast genes. Moreover, at the level of gene expression, we noticed that for those genes whose inactivation was predicted to have a negative impact on fitness, the upregulation of transcription is more often observed than downregulation. We tend to think that the adaptive landscape of compensatory evolution is not stable and evolutionary trajectories are unpredictable using landscapes derived for specific conditions. In contrast, general adaptations to given environmental conditions and the landscape of such adaptations are stable. The results obtained with our model indicate that the modular structure of the cellular machinery has a limited impact on short-term evolution in the conditions assessed.

## Materials and methods

### Strains and media

Haploid *S. cerevisiae* strains used in this study were derivatives of W303 (MAT**a** or MATα {*leu2-3,112 trp1-1 can1-100 ura3-1 ade2-1 his3-11,15*}). All primers used are listed in Additional file 16. To construct knockout strains *nup133*∆ and *cog7Δ*, *KanMX* cassette conferring resistance to G418 was amplified from the appropriate yeast deletion collection haploid strains (Euroscarf, Germany), along with ~ 200 bp of both ATG-upstream and stop codon-downstream DNA for homology. Wild-type strain W303 was transformed using the lithium acetate method [74] and transformants were selected for G418 resistance. To construct strains with deleted *MSH2* gene*, hphMX4* cassette conferring resistance to hygromycin B was amplified from the pAG32 plasmid [75]. Primers contained 45-bp fragments upstream of ATG and downstream of the stop codon for homology. The wild-type and knockout strains were transformed with amplified DNA and transformants were selected for hygromycin B resistance (single mutant *msh2Δ*) or hygromycin B and G418 resistance (double mutants). All knockout strains were verified for correct cassettes integration by PCR and sequencing of PCR-derived fragments. The strains subjected to continuous culture experiments were grown in a complete synthetic liquid medium containing 0.67% yeast nitrogen base (YNB), 2% glycerol, 0.1% yeast extract, 0.1% glucose and supplemented with the required amino acids and nucleotides. Ampicillin and streptomycin (25 μg/mL) were added to prevent bacterial contamination. For overnight liquid cultures and 2.0% agar–agar solidified plates YPD medium (1% Bacto-yeast extract, 2% Bacto-peptone and 2% glucose) was used.

### Continuous culture evolution experiments

The evolutionary experiments were conducted in a self-made continuous culture set based on [76]. Briefly, the chambers, containing 20 mL of a medium, were made of 50-mL plastic Falcon tubes closed with a silicone stopper pierced with three needles of different lengths. The shortest needle was used to add medium to the chamber. The longest needle reaching to the bottom of the tube provided filtered air (pumped with an aquarium pump) and allowed efficient mixing of cultures. The third needle was used for determining culture volume by removing effluent to collection bottles. To start the experiment 15 mL of liquid YPD medium was inoculated with a single colony and grown overnight at 30°. Aliquots of the overnight cultures were inoculated into growth chambers to OD_600_ = 0.1. After ca. 40 h, the flow of medium was turned on at a dilution rate of 0.17–0.18 vol/h. Each strain was cultivated in triplicate. In total, 18 chambers were inoculated. Culture samples were passively collected every week from fresh effluent for ca. 200 generations (non-mutator strains) or for ca. 350 generations (mutator strains) and stored as dry cell pellets and glycerol stocks at -80°. Throughout the experiment, vessels were monitored for contamination and formation of flocculants. An experiment was terminated when cell clumps precluded efficient mixing of the culture.

### Whole-genome population sequencing (WGS) and sequence analysis

Population genomic DNA was extracted from dry, frozen cell pellets using Bacterial & Yeast Genomic DNA Purification Kit (EurX). DNA quality and quantity were checked by agarose gel electrophoresis and fluorimetric measurements with Qubit dsDNA BR Assay Kit (Thermo Fisher Scientific). Then the DNA was mechanically sheared to an appropriate size and used for paired-end library preparation using KAPA Library preparation kit (Roche) following the manufacturer’s instructions and sequenced in the paired-end mode (2 × 300 bp) on a MiSeq (Illumina) instrument. The sequencing was performed at the DNA Sequencing and Oligonucleotide Synthesis Laboratory of the Institute of Biochemistry and Biophysics PAS, Warsaw, Poland. Sequencing data were filtered by quality using fastp toolkit [77]. The average sequence coverage from WGS of the yeast populations was targeted at around 100×. Progenitor strain reads were aligned against the genome sequence of W303 [78] to build a progenitor consensus genome for each genetic background. The reads were mapped to the W303 sequence using BWA-MEM [79] and genome sequence alignment was corrected using Pilon [80] with the “fix all” option to correct all point mutations, indels, gaps and fix local misassemblies. Ten iterations of the above polishing scheme were run. Progenitor consensus genomes were annotated according to their Illumina reads and mapped to the appropriate progenitor genome consensus using BWA-MEM, followed by processing using SAMtools-mpileup [81]. Variant calling was performed using VarScan v2.3.9 [82] with a minimum variant allele frequency threshold set to 50%. From that point the data were handled with Geneious v10.2.6 software (http://www.geneious.com/). De novo mutational events were identified as alterations from the ancestral strains found in the evolved yeast populations. For that, point mutations and small indels calling were performed with the following options: minimum of ten reads and 50% mutation threshold frequency. Finally, mutations unique to the evolved populations were identified and verified by visual examination directly in Geneious. Variations at ambiguous positions in the sequence were ignored. Selected mutations localized to ORFs were validated in populations using PCR with appropriate primer pairs (Additional file 16) and DNA sequencing.

### DNA content analysis by flow cytometry

The DNA content of yeast cells was measured by flow cytometry as previously described [83], with modifications restricting cells aggregations. About 10^7^ cells from initial and final yeast cultures were spun down (19,300 g for 1 min) and subjected to permeabilization and fixation via suspension in 1 ml of chilled (− 20 °C) 80% ethanol (Polmos, Warsaw, Poland). Cells suspensions were held at room temperature for at least 2 h. The fixed cells were then washed twice in FACS buffer [0.2 M Tris–HCl (Sigma-Aldrich) pH 7.4 and 20 mM EDTA (Merck, Darmstadt, Germany)] and incubated at 37 °C for 2 h in FACS buffer with 1 mg/ml RNase A (Sigma-Aldrich). After removal of cellular RNA, cells were washed with sodium citrate buffer (50 mM pH 7.2) and incubated at 55 °C for 1 h in sodium citrate buffer (50 mM pH 7.2) with 2 mg/ml Proteinase K (A&A Biotechnology) to remove proteins. Then, the cells were washed with phosphate buffered saline (PBS) and stained overnight at 4 °C in the dark with 100 μl of propidium iodide solution (50 μg/ml in PBS; Calbiochem). After the addition of 900 μl of PBS, EDTA was added to a final concentration 20 mM, and the cells were sonicated at least three times for 15 s each using Microson XL Ultrasonic Cell Disruptor (Misonix), to avoid cell clumping, just before flow cytometry analysis of the DNA content. This analysis was performed with a FACSCalibur analyzer (Becton–Dickinson, Franklin Lakes, NJ). A total of 10,000 cells in each sample were counted.

### Preparation of total RNA and transcriptome analysis

Total RNA was extracted from frozen yeast cell samples obtained from 5 mL of continuous culture using the acid phenol–chloroform procedure [74]. The quantity and quality of the RNA preparations were tested with the 2100 Bioanalyzer expert assay RNA Nano (Agilent Technologies). Transcriptomes of the evolved cell populations were compared with that of the original one using Yeast (v2) Gene Expression 8 × 15 K Microarray slides (Agilent Technologies) containing oligonucleotides that representing all known *S. cerevisiae* genes identified in the SGD. cDNA probes labeled with Cy3 or Cy5 fluorescent dyes were synthesized using the Agilent Two-Color Quick Amp Labeling Kit according to the manufacturer’s protocol, with total RNA preparations as the template. The labeled probes were hybridized concurrently to microarrays. Three biological replicates were run, each with two technical replicates with dye-swap. The resulting fluorescence images were scanned with an Axon GenePix 4000B (Molecular Devices) microarray scanner. Feature extraction was done with GenePix Pro 6.1. Raw data were normalized, technical replicates were averaged, and subjected to statistical analysis with Acuity 4.0 software. Additional data manipulations were done in Microsoft Excel. For each gene, the log2 ratio of its transcript level in each evolved population to that in the preadapted culture was calculated. Genes displaying log2 ratio ≥ 1 or ≤ − 1 in at least two of three biological replicates for a given cell population genotype were considered differentially expressed (DEGs) and were subjected to further bioinformatic analysis. UpSet diagrams were generated using an Intervene online tool [84]. To classify the functions of the identified DEGs, GO analysis was conducted using a Cytoscape (v3.7.2) plugin BiNGO [85] and GO Slim Term Mapper (https://www.yeastgenome.org/goSlimMapper). Euclidean distances were calculated with the following equation: $\sqrt{{\left({p}_{1}-{q}_{1}\right)}^{2 }+{\left({p}_{2}-{q}_{2}\right)}^{2 }+ \dots +{\left({p}_{n}-{q}_{n}\right)}^{2 }},$ where *p* and *q* are log2 ratios for each gene in two transcriptomes being compared and *n* is the number of genes. Cluster analysis and the similarity tree visualization was done on the whole transcriptome data using Cluster 3.0 [86] and Java TreeView [87] software, respectively.

### Reverse transcription-quantitative PCR (RT-qPCR) analyses

To validate the microarray results the RT-qPCR was performed in triplicate using QUANTUM EvaGreen® PCR Kit (Syngen Biotech) according to the manufacturer’s instructions and a Mic Real-Time Cycler (Bio Molecular Systems). The primers used (Additional file 16) were based on qPrimerDB (version 1.2) data. Primer specificity was verified by melting curve analysis. Each 20 μl reaction mixture contained 4 μl of QUANTUM EvaGreen® PCR Kit mix, two primers (4 pmol each) and 1 μl of template cDNA. The cDNA was synthesized from 240 ng of total RNA treated with TURBO™ DNase (Invitrogen) using smART First Strand cDNA Synthesis kit (EurX) according to supplier’s protocol. The qPCR reactions were carried out at the following conditions: 95° for 15 min, followed by 40 cycles of 95° for 15 s, 51.5° for 20 s and 72° for 20 s. The value of crossing threshold cycles (Cq) was determined using the micPCR software v2.8.10. Pfaffl method [88] was applied to calculate relative expression with respect to that of *ACT1* that was used as the normalization reference for target gene expression*.* Then, the fold difference of the gene expression levels between evolved and starting populations, corrected for efficiency, was calculated.

## Supplementary Information

**Additional file 1.** Doubling times and growth rates of yeast strains studied.

**Additional file 2.** DNA content analysis of the ancestral (START) and evolved (END) yeast populations. Flow cytometry profiles indicate that short-term continuous culturing experiments did not cause changes in ploidy as all yeast populations studied remain haploid. Control haploid and diploid strains are shown on the right.

**Additional file 3.** Mutations in ORFs of evolved non-mutator strains, grouped according to their predicted effect.

**Additional file 4.** Mutations in ORFs of evolved mutator strains, grouped according to their predicted effect.

**Additional file 5.** Detailed description of mutations identified in evolved yeast strains.

**Additional file 6.** Extended characteristics of nonsynonymously mutated genes of evolved cog7Δ msh2Δ strain.

**Additional file 7.** Extended characteristics of nonsynonymously mutated genes of evolved nup133Δ msh2Δ strain.

**Additional file 8.** The similarities of the adaptive transcriptomes of all biological replicates of continuous cultures performed in this study, expressed as Euclidean distance parameter and similarity tree obtained after clustering of all transcriptome data.

**Additional file 9.** Microarray data on all evolved yeast populations subjected to analysis.

**Additional file 10.** Comparison of changes in transcript levels determined by microarray hybridization with RT-qPCR quantification for selected genes.

**Additional file 11.** Number of DEGs for each biological replicate and total number of DEGs for each evolved yeast strain.

**Additional file 12.** Correlation of point mutations located to ORF, and putative promoter and terminator regions and transcriptomic changes in evolved yeast strains.

**Additional file 13.** UpSet diagrams for genes whose expression was significantly up- (red) and downregulated (blue) in the three evolved biological replicates of given yeast genotype.

**Additional file 14.** Top enriched GO terms of differentially expressed genes for all strains studied. Only the enriched GO terms of “biological process” and “cellular component” categories were analyzed.

**Additional file 15.** Comments to Additional file 14.

**Additional file 16.** Primers used in this study.

## Data Availability

All strains are available upon request. The authors affirm that all data necessary for confirming the conclusions of the article are present within the article and its additional files. Complete WGS data have been deposited in the GenBank under the BioProject ID PRJNA694549. Complete transcriptome analysis data are deposited in the Gene Expression Omnibus (https://www.ncbi.nlm.nih.gov/geo) under accession number GSE167397.
