# Supplementary material for: Genetic interaction network has a very limited impact on the evolutionary trajectories in continuous culture-grown populations of yeast
Source: BMC Ecol Evol. 2021 May 26;21:99. doi: 10.1186/s12862-021-01830-9 (PMC8157726; doi:10.1186/s12862-021-01830-9)
Supplement: Supplementary file 1 — Additional file 1. Doubling times and growth rates of yeast strains studied. [file 12862_2021_1830_MOESM1_ESM.docx]

| **Strain** | **Doubling time [min]** | **Increase in doubling time [%]** | **Relative growth rate [%]** |
| --- | --- | --- | --- |
| WT | 254 | 0.0 | 100 |
| *cog7Δ* | 255 | 0.4 | -0.4 |
| *nup133Δ* | 314 | 23.6 | -19.1 |
| *msh2Δ* | 270 | 6.3 | -5.9 |
| *cog7Δ msh2Δ* | 273 | 7.5 | -7.0 |
| *nup133Δ msh2Δ* | 311 | 22.4 | -18.3 |

## Additional file 1*.* Doubling times and growth rates of yeast strains studied.

The growth rate and doubling time determination was based on growth curves, measurements were taken at exponential growth. Initially, yeast cultures were grown overnight and then were diluted in the same medium which was used during the evolutionary experiments to optical density (OD_600_) = 0.1. Strains growth in liquid cultures was monitored by OD_600_ measurements. Growth rates (r) were calculated by the formula r = (ln [OD2/OD1]) / (T2-T1) and the doubling time corresponds to ln(2)/r.
