# Supplementary material for: Genetic interaction network has a very limited impact on the evolutionary trajectories in continuous culture-grown populations of yeast
Source: BMC Ecol Evol. 2021 May 26;21:99. doi: 10.1186/s12862-021-01830-9 (PMC8157726; doi:10.1186/s12862-021-01830-9)

**Additional file 2*.*** DNA content analysis of the ancestral (START) and evolved (END) yeast populations. Flow cytometry profiles indicate that short-term continuous culturing experiments did not cause changes in ploidy as all yeast populations studied remain haploid. Control haploid and diploid strains are shown on the right.


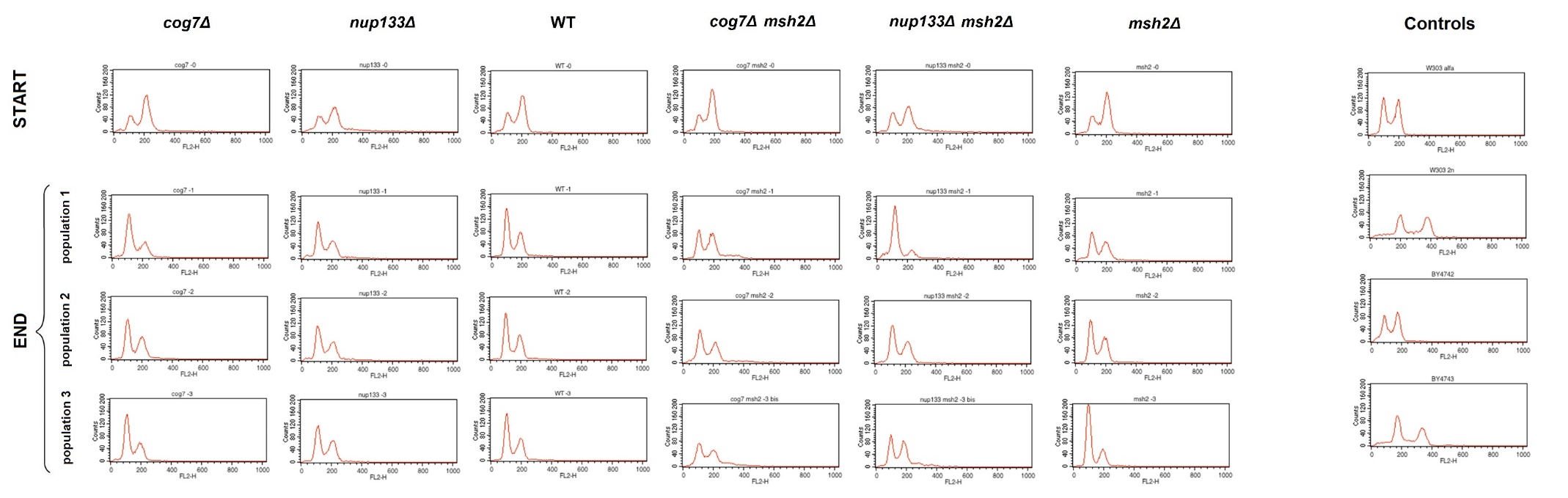

Supplement: Supplementary file 2 — Additional file 2. DNA content analysis of the ancestral (START) and evolved (END) yeast populations. Flow cytometry profiles indicate that short-term continuous culturing experiments did not cause changes in ploidy as all yeast populations studied remain haploid. Control haploid and diploid strains are shown on the right. [file 12862_2021_1830_MOESM2_ESM.docx]
