# Supplementary material for: Genetic interaction network has a very limited impact on the evolutionary trajectories in continuous culture-grown populations of yeast
Source: BMC Ecol Evol. 2021 May 26;21:99. doi: 10.1186/s12862-021-01830-9 (PMC8157726; doi:10.1186/s12862-021-01830-9)
Supplement: Supplementary file 3 — Additional file 3. Mutations in ORFs of evolved non-mutator strains, grouped according to their predicted effect. [file 12862_2021_1830_MOESM3_ESM.docx]

| **Protein effect** | ***cog7Δ*** | ***nup133Δ*** | **WT** |
| --- | --- | --- | --- |
|  | Gene name | | |
| substitution/s | *BUL1*; HOG1*; MEC1* | *BUL1*; CAN1; DAN4*; PFK27; WHI2* | *HOG1*; MSY1* |
| frameshift |  |  | *HOG1** |
| truncation |  | *WHI2* | *URE2* |
| none | *DAN4** |  | *DAL82* |
| * gene also mutated in other strain/s | |  |  |

**Additional file 3.** Mutations in ORFs of evolved non-mutator strains, grouped according to their predicted effect.
