# Supplementary material for: Genetic interaction network has a very limited impact on the evolutionary trajectories in continuous culture-grown populations of yeast
Source: BMC Ecol Evol. 2021 May 26;21:99. doi: 10.1186/s12862-021-01830-9 (PMC8157726; doi:10.1186/s12862-021-01830-9)
Supplement: Supplementary file 4 — Additional file 4. Mutations in ORFs of evolved mutator strains, grouped according to their predicted effect. [file 12862_2021_1830_MOESM4_ESM.docx]

| **Protein effect** | ***cog7Δ msh2Δ*** | ***nup133Δ* *msh2Δ*** | ***msh2Δ*** |
| --- | --- | --- | --- |
|  | Gene name | | |
| substitution/s | *AMF1; AMN1; ART5; DAK2; DMA2; ELM1; EXO5; GLN1; GNA1; HSP104; HUR1; HXK1; ICL1; IRR1; KRE5; MRPS5; NET1; PAB1; PIF1; PMR1; PRO3*; PRP2; RPL4B; SEO1; SHM2; SHP1; SKM1; SOL4; SSN3; TKL1; TRX2; WAR1; YPL247C; YPL277C* | *ACC1; APC5; BRE1; CAN1; CEX1; DMA1; EFT2; EMP65; GIC1; GEX1; HSP60; IBA57; INP1; PEX21; PRK1; RSC1; SAM2; SCW4; SLA2; SWI1; YLR177W* | *BRR2; DRN1; MDN1; PMD1; PRO3*; RPL42B; URE2; YBT1* |
| truncation | *ACE2; ECM21; SAP30* | *MEC3; RIM15; TAH1* | *URE2* |
| none | *CDC3; ISW2; NRD1; PEX7; VHS3; YPS1; YER010C* | *ADY3; BDP1; CDC43; DCK1; EGH1; FLO11; GEX1; RGT2; RIX7; SRB8; SRC1; TIP1; YRB30* |  |
| *gene also mutated in other strain/s | |  |  |

**Additional file 4.** Mutations in ORFs of evolved mutator strains, grouped according to their predicted effect.
