# Supplementary material for: Genetic interaction network has a very limited impact on the evolutionary trajectories in continuous culture-grown populations of yeast
Source: BMC Ecol Evol. 2021 May 26;21:99. doi: 10.1186/s12862-021-01830-9 (PMC8157726; doi:10.1186/s12862-021-01830-9)
Supplement: Supplementary file 8 — Additional file 8. The similarities of the adaptive transcriptomes of all biological replicates of continuous cultures performed in this study, expressed as Euclidean distance parameter and similarity tree obtained after clustering of all transcriptome data. [file 12862_2021_1830_MOESM8_ESM.docx]

**Additional file 8.** The similarities of the adaptive transcriptomes of all biological replicates of continuous cultures performed in this study, expressed as Euclidean distance parameter and similarity tree obtained after clustering of all transcriptome data.


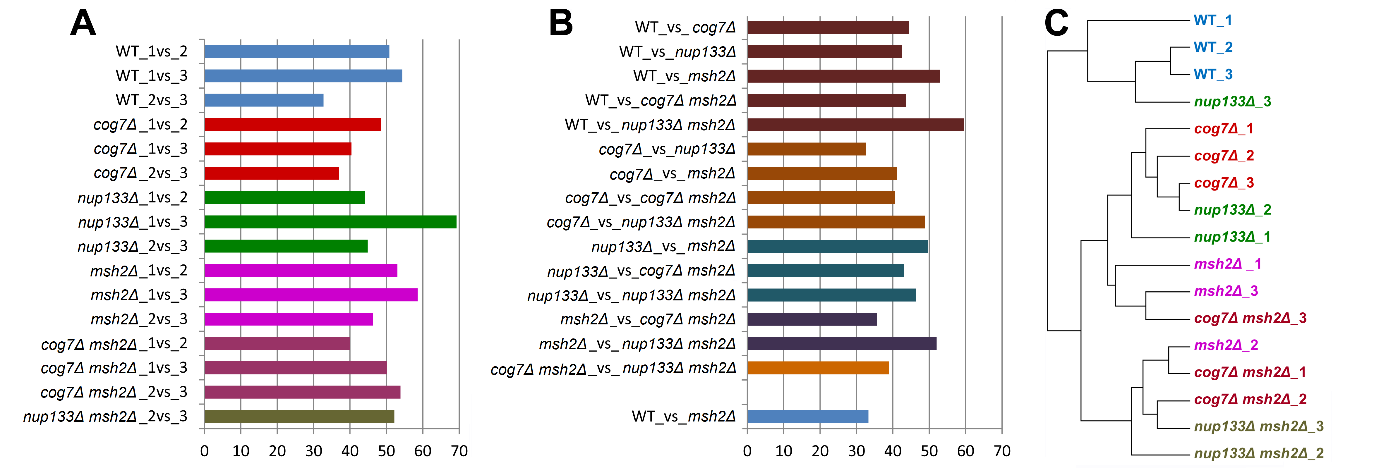


(A) Euclidean distances between adaptive transcriptomes of individual biological replicates of the same genotype (for clarity highlighted with the same color). (B) Euclidean distances between the averaged transcriptome data for different genotypes. (C) Similarity tree obtained after clustering of all transcriptome data (done with Cluster 3.0 and TreeView). Populations evolved from the same initial genotypes are marked with the same color.
