# Supplementary material for: Genetic interaction network has a very limited impact on the evolutionary trajectories in continuous culture-grown populations of yeast
Source: BMC Ecol Evol. 2021 May 26;21:99. doi: 10.1186/s12862-021-01830-9 (PMC8157726; doi:10.1186/s12862-021-01830-9)
Supplement: Supplementary file 11 — Additional file 11. Number of DEGs for each biological replicate and total number of DEGs for each evolved yeast strain. [file 12862_2021_1830_MOESM11_ESM.docx]

|  | Evolved line | Upregulated | | Downregulated | |
| --- | --- | --- | --- | --- | --- |
|  |  | ▲ | Strain-specific DEGs | ▼ | Strain-specific DEGs |
| Non-mutator group | cog7_evo_1 | 411 | 214 | 303 | 161 |
|  | cog7_evo_2 | 336 |  | 307 |  |
|  | cog7_evo_3 | 193 |  | 119 |  |
|  | nup133_evo_1 | 453 | 91 | 456 | 91 |
|  | nup133_evo_2 | 83 |  | 78 |  |
|  | nup133_evo_3 | 141 |  | 205 |  |
|  | WT evo_1 | 445 | 243 | 637 | 239 |
|  | WT evo_2 | 258 |  | 267 |  |
|  | WT evo_3 | 325 |  | 283 |  |
| Mutator group | cog7 msh2 evo_1 | 116 | 112 | 170 | 177 |
|  | cog7 msh2 evo_2 | 232 |  | 182 |  |
|  | cog7 msh2 evo_3 | 402 |  | 420 |  |
|  | nup133 msh2 evo_2 | 306 | 147* | 248 | 124* |
|  | nup133 msh2 evo_3 | 177 |  | 152 |  |
|  | msh2 evo_1 | 729 | 319 | 582 | 271 |
|  | msh2 evo_2 | 197 |  | 264 |  |
|  | msh2 evo_3 | 408 |  | 366 |  |
|  |  |  |  |  |  |
| *due to only 2 of 3 repetitions proceeded - given gene was recognized as DEG (on the strain scale) if its expression has changed at least 2-fold in one of the repetitions and at least 1-fold in the other. | | | | | |

**Additional file 11.** Number of DEGs for each biological repetitions and total number of DEGs for each evolved yeast strains.
