# Supplementary material for: Genetic interaction network has a very limited impact on the evolutionary trajectories in continuous culture-grown populations of yeast
Source: BMC Ecol Evol. 2021 May 26;21:99. doi: 10.1186/s12862-021-01830-9 (PMC8157726; doi:10.1186/s12862-021-01830-9)
Supplement: Supplementary file 13 — Additional file 13. UpSet diagrams for genes whose expression was significantly up- (red) and downregulated (blue) in the three evolved biological replicates of given yeast genotype. [file 12862_2021_1830_MOESM13_ESM.docx]

**Additional file 13.** UpSet diagrams for genes whose expression was significantly up- (red) and downregulated (blue) in the three evolved biological replicates of given yeast genotype.


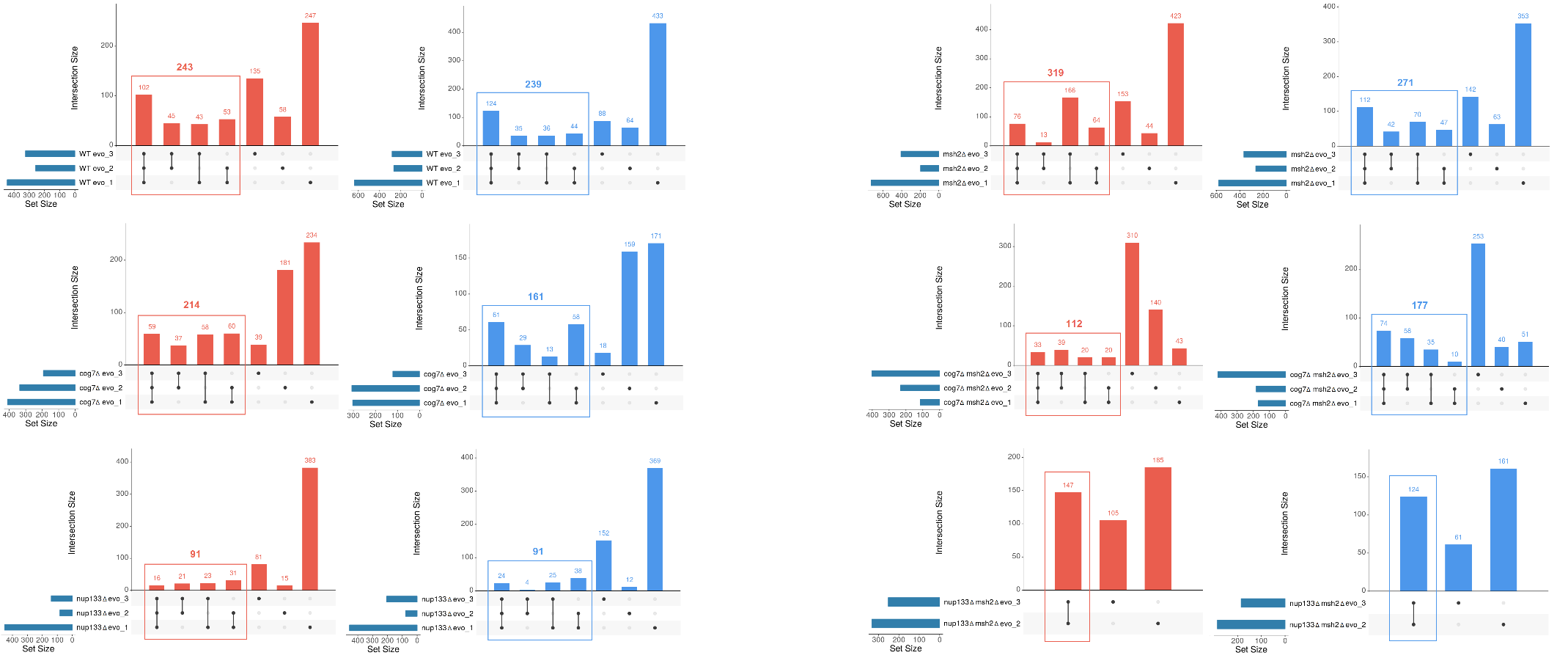


The horizontal bars show the number of differentially expressed genes of a given yeast replicate, while the vertical bars display the size of sets of genes exclusively shared between multiple pairwise comparisons (on the left) followed by those uniquely altered in the given replicate.
