# Supplementary material for: Genetic interaction network has a very limited impact on the evolutionary trajectories in continuous culture-grown populations of yeast
Source: BMC Ecol Evol. 2021 May 26;21:99. doi: 10.1186/s12862-021-01830-9 (PMC8157726; doi:10.1186/s12862-021-01830-9)
Supplement: Supplementary file 15 — Additional file 15. Comments to Additional file 14. [file 12862_2021_1830_MOESM15_ESM.docx]

#### Comments to Additional file 14.

#### Non-mutator populations

Among the upregulated DEGs of the evolved *cog7Δ* strain, the enriched biological process category terms were in ribonucleoprotein complex, ribosomal small and large subunit biogenesis and assembly, and ncRNA processing and modification. In the category of cellular component, the enriched GO terms were related to preribosome (small and large subunit precursors).

Among the downregulated DEGs of the evolved *cog7Δ* strain, the enriched GO terms in the category of the biological process were associated with the amino acid metabolism (14 genes), respiration, organic acid transport, and superoxide metabolism (4 genes: fructose-1,6-bisphosphatase, copper-zinc superoxide dismutase and two metallothioneins involved in a defense reaction associated with oxidative stress and in detoxification of harmful metal ions). This suggests changes in general metabolism and the response to reactive oxygen species. In the category of cellular component, the enriched GO terms were related mainly to the mitochondrion (respiratory chain).

Among the upregulated DEGs of the evolved *nup133Δ* strain, the top enriched GO terms in the biological process category were associated with the metabolism of organic acids, lipids, and amino acids metabolism, suggesting that the DEGs were mainly involved in general metabolism. Interestingly, two genes, *PDH1* and *CIT3* that take part in the 2-methylcitrate cycle (2-MCC) were also present in this set. The 2-methylcitrate cycle involves five enzymes converting propionate to pyruvate. Cit3 is important to the mitochondrial metabolism of propionyl-CoA whereas Pdh1 is a mitochondrial protein that participates in respiration induced by diauxic shift. In the category of cellular component, there was enrichment in genes related to peroxisome (6 genes) and phosphopyruvate hydratase complex (*ERR1* and *ERR2*).

Among the downregulated DEGs of the evolved *nup133Δ* strain, the enriched GO terms in the category of the biological process were mainly associated with cell adhesion (agglutinins: *AGA1*, *SAG1*) and response to chemical stimuli. In the cellular component category, enrichment of genes associated with plasma membrane was found.

Among the upregulated DEGs of the evolved wild-type strain, the top enriched GO terms in the biological process category were associated with amino acids metabolism (e.g., *ASP3* (all four copies), *BAT2, CAR2, GDH2,* and *PUT1*), and fatty acid oxidation. In the category of cellular component, enrichment was found in periplasmic space, cytoplasmic microtubule, and nuclear cohesin complex.

Among the downregulated DEGs of the evolved wild-type strain, the enriched terms in the category of the biological process were mainly associated with amino acid transport (e.g., permeases encoded by *AGP1, BAP3, GNP1, HIP1, TAT1*, and *TAT2*), fungal-type cell wall organization, ion transport, and response to abiotic stimuli, suggesting that most of the downregulated genes were involved in general cellular metabolism. Additionally, we observed a downregulation of genes involved in glycerol synthesis (*GPP1* and *GPP2*), which is in line with expectations since the medium contained glycerol as the main carbon source. Enriched GO terms in the cellular component category were mainly connected with ribosomes and cell wall.

We also checked which GO terms were enriched among genes commonly up- or downregulated shared by non-mutator strains tested. As a result, 81 upregulated genes and 112 downregulated ones were shared by at least two of three non-mutator strains. These DEGs are not related to the compensatory evolution of the mutants but rather reflect the universal adaptation to culture conditions. The upregulated DEG set is enriched in genes involved in organic acids metabolism and the encoded proteins associated with the peroxisome. The shared downregulated genes are enriched in genes linked to transmembrane transport and respiration. In the cellular component category enriched GO terms were linked to genes connected with mitochondrion, and to plasma membrane.

#### Mutator populations (*msh2Δ* bearing)

Among the upregulated DEGs in the evolved *cog7Δ msh2Δ* strain, in the biological process category, enriched were the GO terms linked to amino acids metabolism, peptide transport, and hexose transport (i.e., *HXK1* - engaged in glucose phosphorylation, its expression is highest during growth on non-glucose carbon sources*, HXT3, HXT6,* and *HXT7* - encoding glucose transporters) suggesting that the DEGs were mainly involved in sugar uptake, amino acid metabolism, and other basic metabolic processes. In the cellular component category, mainly enrichment in genes related to the plasma membrane was found.

Among the downregulated DEGs of the evolved *cog7Δ msh2Δ* strain, the GO terms in the category of the biological process were enriched in terms associated e.g., with sulfur amino acid biosynthesis (e.g., *MET3*, *MET5, MET10, MET14, MET17*, and *SER33*) as well as with reproduction, and pyridoxine metabolic process. In the category of cellular component, the enriched GO terms were e.g., linked to sulfite reductase complex, cell wall, and plasma membrane.

Among the upregulated DEGs of the evolved *nup133Δ msh2Δ* strain, the enriched GO terms in the biological process category were associated with carbohydrate transport, formate metabolism (*FDH1, FDH2,* and YPL276W (*FDH2* alias)), cellular amide metabolic process, and peptide transport. In the cellular component category enrichment was found in genes related to the plasma membrane.

Among the downregulated DEGs of the evolved *nup133Δ msh2Δ* strain, the GO terms in the category of the biological process were enriched in those associated with *de novo* IMP (inosine monophosphate) biosynthesis (i.e., *ADE1, ADE2, ADE4, ADE17*), reproduction, and siroheme metabolic process. In the category of cellular component enrichment was found in genes of glycine cleavage complex, extracellular region, and plasma membrane.

It is worth mentioning that the upregulated DEGs of the evolved *msh2Δ* and *cog7Δ* strain were enriched in GO terms belonging to similar processes and cell compartments. In detail, in the evolved *msh2Δ* strain enriched GO terms were associated with RNA 5'-end processing, and rRNA modifications. In the category of cellular component, the enriched GO terms were related to ribosome biogenesis and associated with RNA polymerase complex. In fact, a total of 55 upregulated genes were common to *msh2Δ* and *cog7Δ*. In contrast, *cog7Δ* and *cog7Δ msh2Δ* share only one upregulated gene (Figure 5A in the main text).

Among the downregulated DEGs of the evolved *msh2Δ* strain, the GO terms in the category of the biological process were enriched in those linked to amino acids metabolism, pentose-phosphate shunt, formate metabolism (*FDH1, FDH2,* and YPL276W (*FDH2* alias)), nitrogen utilization (e.g., *ADY2, ATO2, ATO3*) and acetyl-CoA metabolism (e.g., *ICL1, KGD1, KGD2, MDH2, MLS1, SHH4*). In the category of cellular component the enrichment of genes associated with the plasma membrane and cell wall was found.

As previously, we checked the enrichment of GO terms among genes commonly up- or downregulated shared by tested mutator strains. As a result, 83 upregulated, and 119 downregulated genes were shared by at least two of three mutator strains. These DEGs were likely related to the adjustment to continuous culture conditions, as in the case of the DEGs shared by the *MSH2* strains, but also could represent a compensatory adjustment to the effects of the *msh2Δ* defect. Among them, the upregulated genes were enriched e.g., in response to chemicals, meiotic cell cycle, and nucleobase-containing small molecule metabolism, while the downregulated ones - e.g., in genes engaged in cellular amino acid metabolism, conjugation, and cell wall organization or biogenesis. In the cellular component category among the upregulated DEGs we observed overrepresentation of genes linked to plasma membrane and vacuole, and among the downregulated DEGs of genes connected to the mitochondrion, plasma membrane, and endoplasmic reticulum.

Interestingly, in terms of common DEGs, non-mutator and mutator strains shared 8.5% (14 DEGs) of upregulated genes and almost 22% (50 DEGs) of downregulated ones.
