# Supplementary material for: Genetic interaction network has a very limited impact on the evolutionary trajectories in continuous culture-grown populations of yeast
Source: BMC Ecol Evol. 2021 May 26;21:99. doi: 10.1186/s12862-021-01830-9 (PMC8157726; doi:10.1186/s12862-021-01830-9)
Supplement: Supplementary file 16 — Additional file 16. Primers used in this study. [file 12862_2021_1830_MOESM16_ESM.docx]

| **Additional file 16.** Primers used in this study. | | | | | |
| --- | --- | --- | --- | --- | --- |
|  |  | |  | | |
| Construction of mutants and verification of correct cassettes integration | | | | | |
| **Primer name** | **Targeted PCR product** | | | **Primer sequence (5'→3')** | |
| NUP133F | *NUP133* or flanked *KanMX* | | | CGATCCTTAGCTCATCGC | |
| NUP133R |  |  |  | GATGAGGATGAAGATCTGG | |
| COG7F | *COG7* or flanked *KanMX* | | | GAGGTGCTTGAAGTTGG | |
| COG7R |  |  |  | CTATCTGAAATAGCATGTCC | |
| MSH2U TEF | flanked *hphMX4* | | | CTTTATCTGCTGACCTAACATCAAAATCCTCAGATTAAAAGTATGAGATCTGTTTAGCTTGCC | |
| MSH2L TEF |  |  |  | ATTATCTATCGATTCTCACTTAAGATGTCGTTGTAATATTAATTATTCGAGCTCGTTTTCGACAC | |
| MSH2F | *MSH2* or flanked *hphMX4* | | | GCCAAAGACAAGTTCCGCACTCC | |
| MSH2R |  |  |  | ACCGGAGATACTCTTTCCAGTGGT | |
| AL1 | internal *KanMX* reverse primer | | | CGTGATTGCGCCTGAGCGAG | |
|  |  | | |  | |
|  |  | | |  | |
| Sanger validation of Illumina WGS results | | | | | |
| **Primer name** | **Targeted PCR product length (bp)** | | | **Primer sequence (5'→3')** | |
| MEC1F | 629 | | | CATGTGTCGAATTTTACGGA | |
| MEC1R |  |  |  | AGCTTTATTAGTCAACTGCG | |
| WHI2F | 768 | | | GAAAAGGGGTCCAATTCTTC | |
| WHI2R |  |  |  | TATATACACTCACTGCACCC | |
| ACC1F | 611 | | | CTATCATCTTCATGGTCCACTG | |
| ACC1R |  |  |  | GAAGTCTTGAAGTATGGTTCGT | |
| BRE1F | 587 | | | TGTAGGGCATTTTCTCATTCTC | |
| BRE1R |  |  |  | CGATGAGGTCCAAAGATTCAAT | |
| CEX1F | 612 | | | TCCACATTTTATCCAAGGTCTG | |
| CEX1R |  |  |  | GCTGAGGGAGAATTTAAACGTA | |
| PMR1F | 566 | | | TCCGGAAAAAGAAGGAAAGA | |
| PMR1R |  |  |  | CCGAAGATGGTAAAGTAACTGC | |
| RSC1F | 779 | | | GGTATTAGCGAAGGGAAAATCT | |
| RSC1R |  |  |  | GGTAGATCAATTACTGGTGGTC | |
| TRX2F | 459 | | | ACATGATGTACTTTACGTAGCG | |
| TRX2R |  |  |  | AAGCTGACAAGAGAATAACGAG | |
| BUL1F | 610 | | | CCCGACGTATACTCTTTCAA | |
| BUL1R |  |  |  | AACTAGCACATTCTCATTCG | |
|  |  | | |  | |
|  |  | | |  | |
| RT-qPCR validation of microarray results | | | | | |
| **Primer name** | | **Targeted PCR product** | | | **Primer sequence (5'→3')** |
| ACT1F | | *ACT1* (reference) | | | AGAGTTGCCCCAGAAGAACA |
| ACT1R | |  |  |  | GGCTTGGATGGAAACGTAGA |
| L_YLR413W_1150-1449 | | *INA1* | | | TGACAACTATCACACCAGAACA |
| R_YLR413W_1150-1449 | |  |  |  | TTGTTCTGTTTAAGTGAGCACG |
| L_YJR004C_350-649 | | *SAG1* | | | GATGTGGTGAATTTCGATCCTG |
| R_YJR004C_350-649 | |  |  |  | TCAATCTTCTCAGTACCACCC |
| L_YKR026C_0-299 | | *GCN3* | | | ATACTGAGATGACAATGCCGAT |
| R_YKR026C_0-299 | |  |  |  | GAAAATATCACAACCGGCTCTC |
| L_YHR196W_50-349 | | *UTP9* | | | CTTTGGGTGGTGTAGTGATTTG |
| R_YHR196W_50-349 | |  |  |  | AAACAACAATTCTTCCGTCAGG |
| L_YOL104C_400-699 | | *NDJ1* | | | CCGTTTTAACAAAATTTGGCCC |
| R_YOL104C_400-699 | |  |  |  | CATTCGGAATGTTCTGATGGAC |
| L_YER126C_400-699 | | *NSA2* | | | CACTCATCCGGAATTAGGTGTA |
| R_YER126C_400-699 | |  |  |  | CCCAATTCCGAAACGTTAACTT |
| L_YHR065C_600-899 | | *RRP3* | | | GACATGGAATTTGGACCTGTTC |
| R_YHR065C_600-899 | |  |  |  | ACAGGATTTGTCAAACTTGCTC |
| L_YHR088W_50-349 | | *RPF1* | | | CACTAGAGTGTACGATGAGACC |
| R_YHR088W_50-349 | |  |  |  | TTTGGTGGCTCATTAGAATTGC |
